# Three separate pathways in *Rhizobium leguminosarum* maintain phosphatidylcholine biosynthesis, which is required for symbiotic nitrogen fixation with clover

**DOI:** 10.1128/aem.00590-24

**Published:** 2024-08-09

**Authors:** Julia Kleetz, Ann-Sophie Mizza, Irina Shevyreva, Leon Welter, Claudia Brocks, Anja Hemschemeier, Meriyem Aktas, Franz Narberhaus

**Affiliations:** 1 Microbial Biology, Faculty of Biology and Biotechnology, Ruhr University Bochum, Bochum, Germany; 2 Photobiotechnology, Faculty of Biology and Biotechnology, Ruhr University Bochum, Bochum, Germany; Georgia Institute of Technology, Atlanta, Georgia, USA

**Keywords:** bacterial membrane, host-microbe interaction, nitrogen fixation, phospholipids, phosphatidylcholine, *Rhizobium*

## Abstract

Phosphatidylcholine (PC) is critical for the nitrogen-fixing symbiosis between rhizobia and legumes. We characterized three PC biosynthesis pathways in *Rhizobium leguminosarum* and evaluated their impact on nitrogen fixation in clover nodules. In the presence of choline, a PC synthase catalyzes the condensation of cytidine diphosphate-diacylglycerol with choline to produce PC. In the presence of lyso-PC, acyltransferases acylate this mono-acylated phospholipid to PC. The third pathway relies on phospholipid *N*-methyltransferases (Pmts), which sequentially methylate phosphatidylethanolamine (PE) through three rounds of methylation, yielding PC via the intermediates monomethyl-PE and dimethyl-PE. In *R. leguminosarum*, at least three Pmts participate in this methylation cascade. To elucidate the functions of these enzymes, we recombinantly produced and biochemically characterized them. We moved on to determine the phospholipid profiles of *R. leguminosarum* mutant strains harboring single and combinatorial deletions of PC biosynthesis genes. The cumulative results show that PC production occurs through the combined action of multiple enzymes, each with distinct substrate and product specificities. The methylation pathway emerges as the dominant PC biosynthesis route, and we pinpoint PmtS2, which catalyzes all three methylation steps, as the enzyme responsible for providing adequate PC amounts for a functional nitrogen-fixing symbiosis with clover.

**IMPORTANCE:**

Understanding the molecular mechanisms of symbiotic nitrogen fixation has important implications for sustainable agriculture. The presence of the phospholipid phosphatidylcholine (PC) in the membrane of rhizobia is critical for the establishment of productive nitrogen-fixing root nodules on legume plants. The reasons for the PC requirement are unknown. Here, we employed *Rhizobium leguminosarum* and clover as model system for a beneficial plant-microbe interaction. We found that *R. leguminosarum* produces PC by three distinct pathways. The relative contribution of these pathways to PC formation was determined in an array of single, double, and triple mutant strains. Several of the PC biosynthesis enzymes were purified and biochemically characterized. Most importantly, we demonstrated the essential role of PC formation by *R. leguminosarum* in nitrogen fixation and pinpointed a specific enzyme indispensable for plant-microbe interaction. Our study offers profound insights into bacterial PC biosynthesis and its pivotal role in biological nitrogen fixation.

## INTRODUCTION

The gram-negative soil bacterium *Rhizobium leguminosarum* has been thoroughly studied for its ability to form symbiotic relationships with legumes. Upon infection of the host plant, nodules are formed in which bacteria differentiate into bacteroids. These bacteroids can convert atmospheric nitrogen into ammonia through nitrogen fixation, thereby providing a crucial nitrogen source for the host plant. Based on the preferred symbiotic interaction partner of *R. leguminosarum*, its species can be further divided into three biovars (1, 2). *R. leguminosarum* bv. *viciae* forms nitrogen-fixing nodules with *Pisum* (pea) or *Vicia* (vetch), bv. *phaseoli* interacts with *Phaseolus* spp. (bean), and bv. *trifolii* with *Trifolium* spp. (clover) (3–7). The host range is primarily determined by the secretion of specific flavonoids by the plant, which upon perception regulate nodulation (*nod*) genes in the bacterium. Conversely, Nod factors produced by the rhizobia are also crucial determinants of host range (8–11). Additional factors affecting the symbiosis of *R. leguminosarum* include the production and composition of exopolysaccharides and lipopolysaccharides as well as the presence of unusually long and multi-unsaturated fatty acyl chains, indicating the significant role of cell surface properties in host interaction (12–16).

In various rhizobia species, the phospholipid composition, particularly the presence of phosphatidylcholine (PC), plays a pivotal role in bacterium-host interactions [for reviews see references (17) and (18)]. For instance*, Bradyrhizobium diazoefficiens,* formerly known as *Bradyrhizobium japonicum* (19)*,* is a nodule-forming and nitrogen-fixing symbiont of soybeans. Mutants of *B. diazoefficiens* exhibiting only 10%–15% PC levels compared to the wild type produced nodules inefficient in nitrogen fixation (20–22). Interestingly, *Bradyrhizobium* sp. SEMIA 6144 showed functional nodules with a 50% reduction in PC levels, suggesting the existence of a threshold of PC necessary for effective nitrogen fixation (23). A puzzling case is a *Bradyrhizobium* toxin-antitoxin mutant that produces incomplete lipopolysaccharides and lacks PC, but nonetheless forms about two-thirds of the nodules that the wild type forms (22). How effective these nodules are in nitrogen fixation activity has not been analyzed. In the absence of PC, *Sinorhizobium meliloti,* the symbiont of alfalfa, fails to form nitrogen-fixing nodules (24, 25). Interestingly, PC does not only play a role in rhizobia-legume interactions, but it is also crucial for tumor formation by the plant pathogen *Agrobacterium tumefaciens (26*), underscoring the significance of PC in both mutualistic and pathogenic plant-microbe interactions.

Notably, the zwitterionic phospholipid PC is not commonly found in bacterial membranes. It has been estimated that approximately 15% of bacterial species have the capability to synthesize PC (17). Remarkably, a significant proportion of these PC-synthesizing species engages in interactions with eukaryotic hosts. In addition to the plant-interacting species introduced above, also the human pathogens *Brucella abortus* and *Legionella pneumophila* produce PC, and mutants deficient in PC production exhibit various degrees of attenuated virulence (27–29).

Bacteria utilize three pathways for PC production, with the methylation and the PC synthase pathway being the primary ones, while the acylation pathway has been described in only a few bacteria (30). In the latter, glycerophosphocholine (GPC) is first acylated to lyso-PC (LPC) and then further to PC using acyl-CoA as acyl donor. In the PC synthase pathway, the ubiquitous phospholipid precursor cytidine diphosphate diacylglycerol (CDP-DAG) is condensed with choline, a reaction catalyzed by the PC synthase (Pcs). On the other hand, the methylation pathway involves a three-step *N*-methylation of phosphatidylethanolamine (PE) utilizing *S*-adenosylmethionine (SAM) as methyl donor. This pathway yields the intermediates monomethyl-PE (MMPE) and dimethyl-PE (DMPE), ultimately leading to the production of PC. The methylation reactions are catalyzed by phospholipid *N*-methyltransferases (Pmts). Bacterial Pmts can be categorized into two main groups based on their amino acid sequence homology. Pmts resembling the enzyme found in *S. meliloti* are classified as S*-*type, while those similar to *Rhodobacter sphaeroides* PmtA belong to the R*-*type. Certain Pmts, such as those from *A. tumefaciens, R. sphaeroides,* and *Rubellimicrobium thermophilum*, catalyze the complete methylation process from PE to PC (31–34). In contrast, other Pmts only catalyze specific methylation steps. For instance, Pmts from *Xanthomonas campestris* and various thermophilic actinobacteria produce MMPE, and some also produce DMPE but not PC (30, 34). The underlying structural and functional determinants of these differences in substrate and product preferences among Pmt enzymes remain elusive. The situation becomes even more complicated when multiple Pmt paralogs exist within a single bacterium. In *B. diazoefficiens*, for instance, at least four distinct Pmts with varying substrate specificities have been identified (20). Their biochemical characterization was not possible due to the poor solubility of the purified recombinant enzymes.

*R. leguminosarum* is another plant-beneficial *Rhizobium* species with a complex membrane lipid profile. The membrane of *R. leguminosarum* bv. *trifolii* ANU843 in late exponential growth phase is characterized by substantial quantities of methylated PE derivatives. While PE constitutes 15% of the entire membrane composition, MMPE (47%) is the dominant phospholipid in this bacterium (35). DMPE and PC are present in lesser amounts at 9% and 2%, respectively. The biosynthetic enzymes accountable for this unusual membrane composition are largely unexplored. The presence of MMPE and DMPE strongly suggests an active methylation pathway. Indeed, studies using radiolabeled SAM have detected methylated PE derivatives in cell-free *R. leguminosarum* extracts (36), and sequence homology searches have uncovered one R-type and two S-type Pmt candidates (37), along with a Pcs candidate (17, 36). The principle aim of this study was to understand the mechanisms underlying the complex PC biosynthesis in *R. leguminosarum*. Through a comprehensive combination of *in silico*, *in vivo,* and *in vitro* methodologies, we identified and characterized three distinct pathways responsible for the formation of methylated PE lipids in *R. leguminosarum* bv. t*rifolii* ATCC14479. Finally, we demonstrated the indispensability of PC for symbiotic nitrogen fixation in clover plants and narrowed down the Pmt enzyme primarily responsible for effective symbiosis.

## RESULTS

### Identification of putative PC biosynthesis enzymes in *R. leguminosarum*

**Fig 1:**
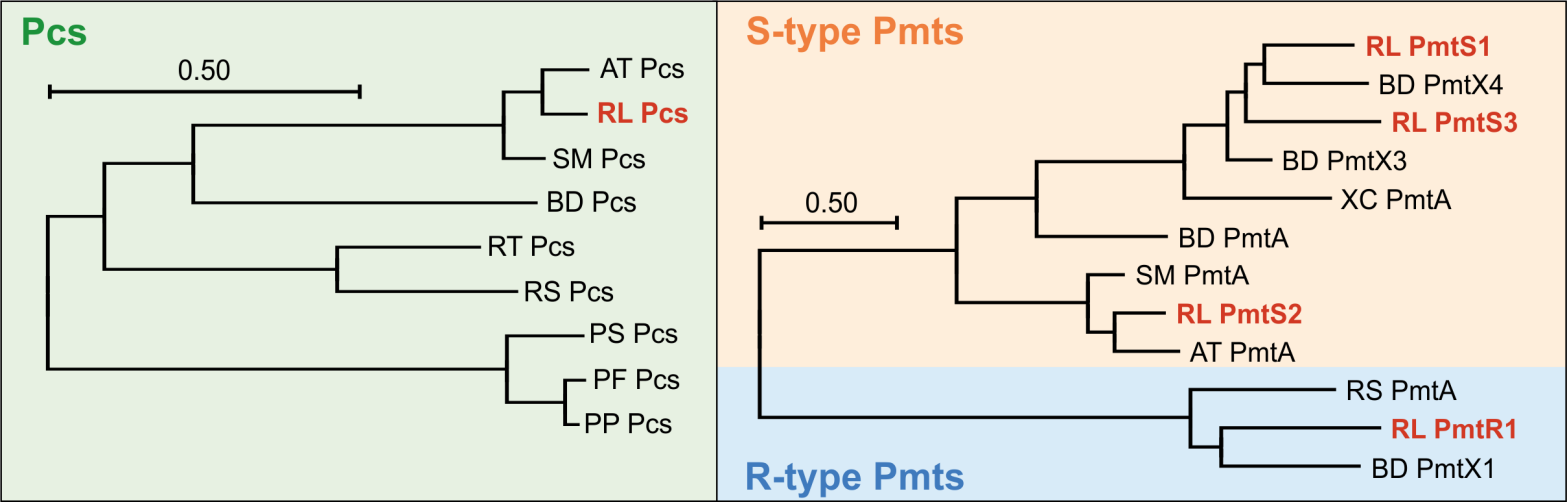
Phylogenetic comparison of PC biosynthesis enzymes identified in *R. leguminosarum*. All trees were constructed using the MegaX software (39). Evolutionary relationships were predicted using the maximum likelihood method (40) and are based on a multiple sequence alignment of amino acid sequences created using the clustalW algorithm (41). Scale bars represent average substitutions per site. Proteins from *R. leguminosarum* are highlighted in red. NCBI identification numbers: *R. leguminosarum* (RL) PmtS1 (DLJ82_3449), *B. diazoefficiens* (BD) PmtX4 (BAC50069), RL PmtS3 (DLJ82_7045), BD PmtX3 (BAC53431), *X. campestris* (XC) PmtA (CAP49368), BD PmtA (BAC45946), *S. meliloti* (SM) PmtA (Q92SN7), RL PmtS2 (DLJ82_3306), *A. tumefaciens* (AT) PmtA (AAK86115), *R. sphaeroides* (RS) PmtA (Q05197), RL PmtR1 (DLJ82_4359), BD PmtX1 (BAC52259), AT Pcs (A9CIM3), RL Pcs (DLJ82_0551), SM Pcs (Q9KJY8), BD Pcs (Q89LF9), *R. thermophilum* (RT) Pcs (S9SEK2), RS Pcs (Q3J0E6), *Pseudomonas syringae* (PS) Pcs (S3MBX7), *Pseudomonas fluorescens* (PF) Pcs (A0A5E6Y2A7), and *Pseudomonas putida* (PP) Pcs (A0A0M3CZZ5).

To identify enzymes potentially involved in PC biosynthesis in *R. leguminosarum*, amino acid sequences of previously described enzymes from different organisms were used as queries for tblastn homology searches (38). A close Pcs homolog was identified using *A. tumefaciens* Pcs as a template (Fig. 1, left panel).

Our search revealed four potential Pmt candidates (Fig. 1, right panel). PmtR1 was identified with *R. sphaeroides* Pmt (R-type) serving as the reference template. PmtS1 and PmtS2 were identified as potential S-type Pmts utilizing *S. meliloti* PmtA as the query. Although previous bioinformatic analyses had suggested the presence of three Pmts in total (37), we identified a fourth Pmt candidate (PmtS3) using *B. diazoefficiens* PmtX3 as the query sequence. Unlike all other identified PC biosynthesis genes, the *pmtS3* gene is situated not on the chromosome but on one of the four plasmids of *R. leguminosarum* ATCC 14479.

### Activity and substrate spectrum of *R. leguminosarum* Pcs in *E. coli*

**Fig 2:**
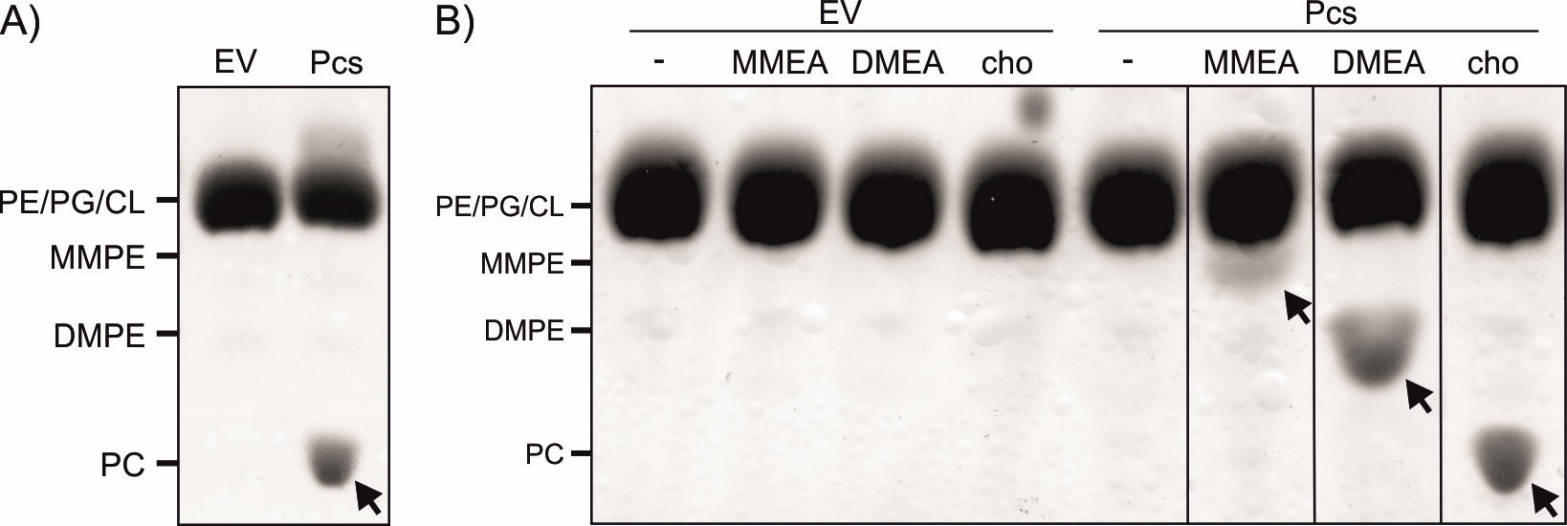
Activity of *R. leguminosarum* Pcs upon heterologous production in *E. coli* BL21 (DE3). Cells carrying the pET28b empty vector (EV) or the *pcs* expression plasmid (Pcs) were grown in LB medium (**A**) or M9 minimal medium (**B**). Gene expression was induced with 0.1 mM isopropyl-*β*-D-thio-galactopyranoside (IPTG), and after incubation of 20 h at 30°C, the cultures were harvested by centrifugation. Lipids were extracted and separated by TLC, and phospholipids were visualized using CuSO_4_. The lipid profile of *E. coli* BL21 (DE3) cells carrying the EV served as negative control. (**B**) For testing the acceptance of different substrates by Pcs, 1 mM of monomethylethanolamine (MMEA), dimethylethanolamine (DMEA), or choline (cho) was added to the cultures simultaneous to expression induction by IPTG. The positions of commercially available C18:1 phospholipids are indicated: phosphatidylethanolamine, phosphatidylglycerol (PG), cardiolipin (CL), monomethyl-PE, dimethyl-PE, and phosphatidylcholine. Arrows highlight formed products.

To demonstrate that the putative Pcs homolog catalyzes the condensation of CDP-DAG with choline to generate PC, the corresponding coding sequence was cloned into a pET28b expression plasmid and expressed heterologously in *E. coli* BL21 (DE3). Subsequently, the lipids of the expression strain were isolated and subjected to thin-layer chromatography (TLC) analysis. *E. coli* cells harboring the empty vector lacked PC, DMPE, and MMPE under any of the tested conditions (Fig. 2A and B). In contrast, the *pcs* expression strain produced PC when cultured in Luria-Bertani (LB) medium (Fig. 2A). While the complex medium contains choline derived from yeast extract, this substrate for Pcs-mediated PC biosynthesis is absent in M9 minimal medium. As expected, *pcs* expression failed to induce PC production in this medium. However, PC formation was restored upon choline supplementation (Fig. 2B).

As reported previously, Pcs enzymes can utilize monomethyl- and dimethylethanolamine (MMEA and DMEA) as substrates, leading to the production of MMPE and DMPE, respectively (42–44). *R. leguminosarum* Pcs exhibited similar substrate flexibility, effectively converting these methylated substrates into MMPE and DMPE (Fig. 2B). Choline and DMEA are favored over MMEA as substrates for Pcs.

### Activity and substrate spectrum of *R. leguminosarum* Pmts in *E. coli*

**Fig 3:**
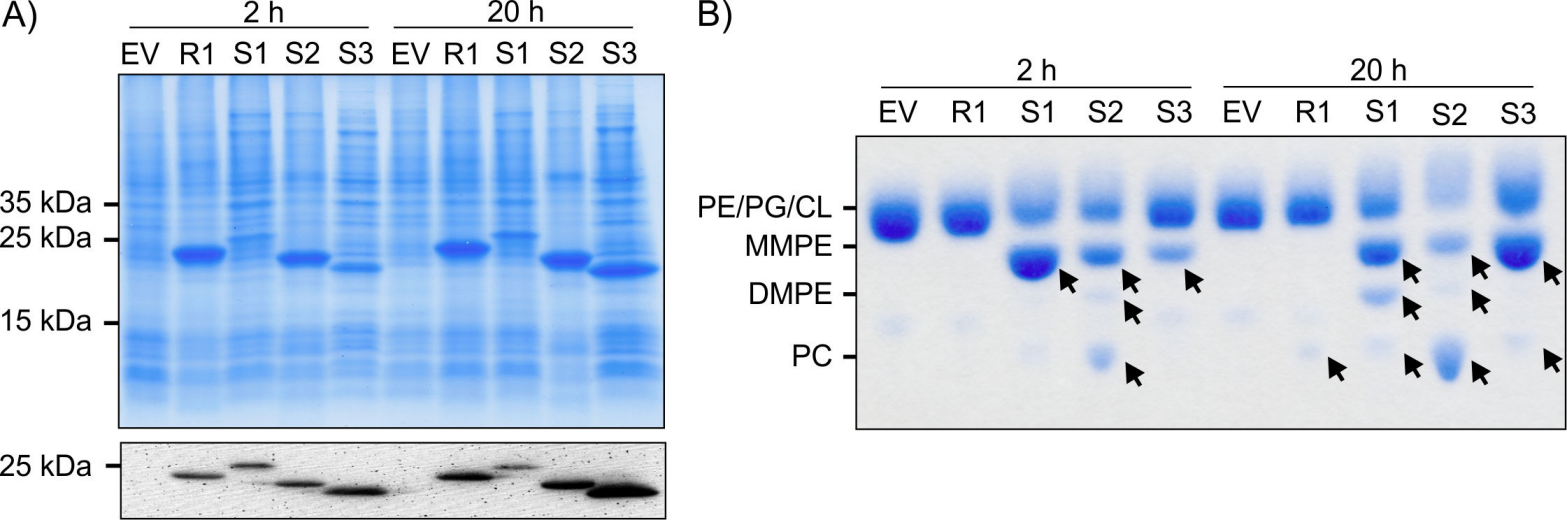
Protein synthesis and activity of the different *R. leguminosarum* Pmts in *E. coli*. All *pmt* genes were expressed in *E. coli* BL21 (DE3) using the respective pET28 expression plasmid. Protein production was induced with 0.1 mM isopropyl-*β*-D-thio-galactopyranoside followed by incubation at 30°C. After 2 and 20 h, cells were harvested for protein and lipid analysis. (**A**) Recombinant protein production was verified by SDS-PAGE and Western blotting using Coomassie blue staining and His-tag-specific antibodies, respectively. (**B**) For analysis of the *E. coli* phospholipids after gene expression, the lipids were extracted, separated by TLC, and subsequently stained with molybdenum blue spray reagent. The position of commercially available C18:1 phospholipids is indicated. Arrows highlight formed products. The protein and lipid profile of *E. coli* carrying the pET28b empty vector (EV) served as negative control. PmtR1 (R1); PmtS1 (S1); PmtS2 (S2); PmtS3 (S3). CL, cardiolipin; PG, phosphatidylglycerol.

To assess the activity of the four Pmt candidates from *R. leguminosarum*, their sequences were cloned into pET28b or pET24b (Table S1) vectors and expressed in *E. coli* BL21 (DE3). The successful production of the recombinant proteins was confirmed, and their correct molecular masses, ranging between 23 and 25 kDa, were validated by sodium dodecyl sulfate polyacrylamide gel electrophoresis (SDS-PAGE) and Western blotting by virtue of the N- or C-terminal His-tags (Fig. 3A).

As shown in Fig. 2, *E. coli* is incapable of synthesizing MMPE, DMPE, or PC in standard laboratory media. However, the substantial presence of PE in the membrane can serve as a substrate for recombinant Pmt enzymes, rendering *E. coli* an appropriate host for analyzing PE-methylating Pmts. Upon induction, overproduction of PmtR1 did not result in the formation of methylated PE derivates after 2 h, although traces of PC were detected after 20 h (Fig. 3B). In contrast, PmtS1 produced copious amounts of MMPE within 2 h of expression. After 20 h, some DMPE and traces of PC were observed, suggesting that PmtS1 primarily catalyzes the initial methylation step and, albeit less efficiently, the subsequent methylation steps. PmtS2 displayed efficient catalysis of all three methylation reactions, yielding the highest amount of PC along with lesser methylated intermediates MMPE and DMPE. Like PmtS1, PmtS3 appeared to primarily catalyze the initial methylation step from PE to MMPE.

### Purification and *in vitro* activity of *R. leguminosarum* Pmts

Given the absence of MMPE and DMPE substrates in *E. coli*, the activities of Pmts methylating these substrates could not be tested in the heterologous host. Hence, all Pmts (except for PmtS3, which could not be purified in an active state) were purified and evaluated for their activity *in vitro*. Commercially available PE, MMPE, and DMPE substrates were employed to assess the enzymatic activity of the purified enzymes.

**Fig 4:**
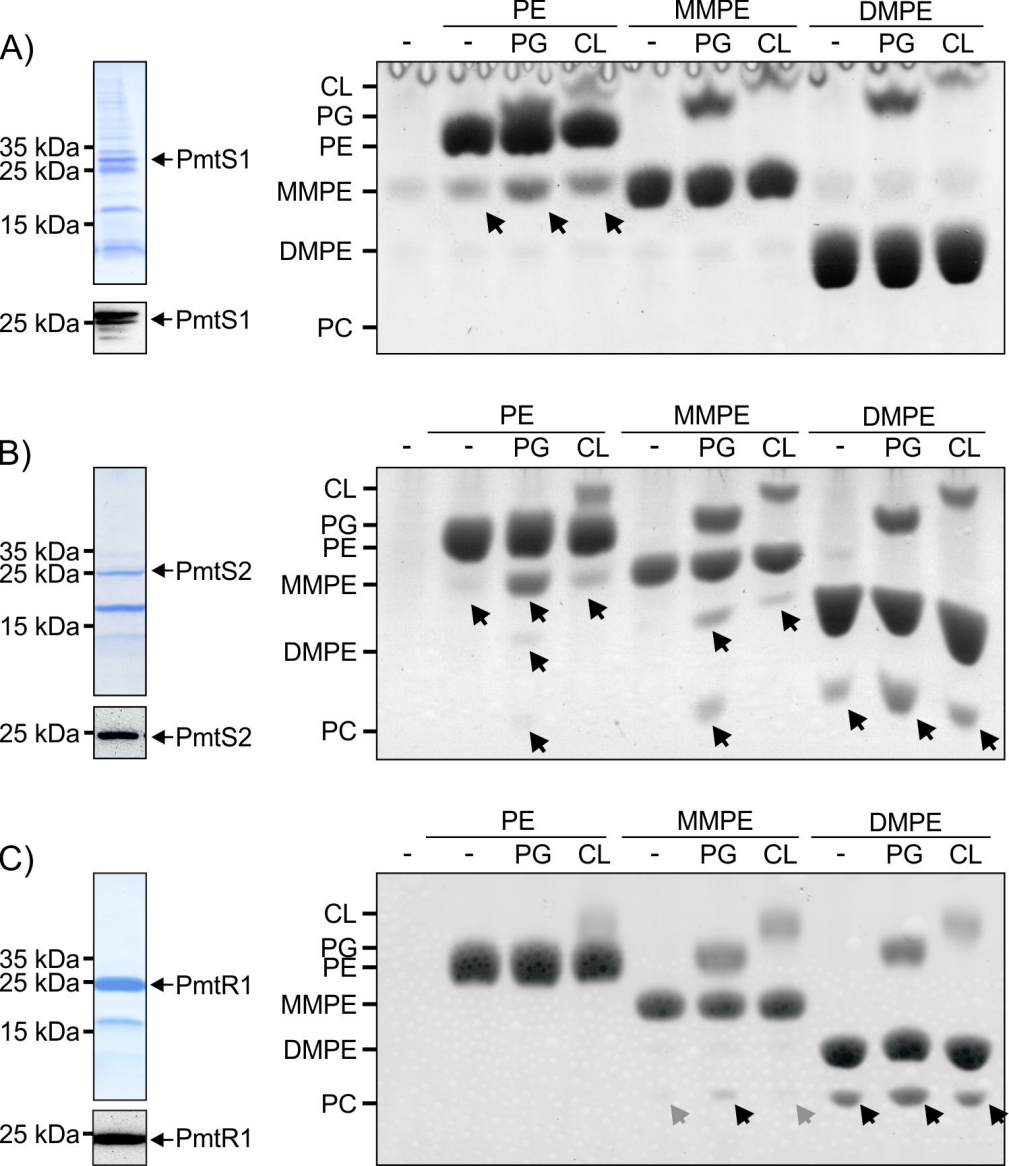
Purification and *in vitro* activity of *R. leguminosarum* Pmts. His-tagged Pmts were produced in *E. coli* BL21 (DE3) using the respective pET24b (PmtS1 and PmtR1) or pET28b (PmtS2) expression plasmid. Recombinant proteins were purified by affinity chromatography (Ni-IDA) (PmtS1; A) followed by size exclusion chromatography (PmtS2 and PmtR1; B and C). The purification steps were monitored by SDS-PAGE and Western blotting using His-tag-specific antibodies. The *in vitro* activity of purified Pmts was analyzed with different lipid substrates and in the presence and absence (−) of anionic lipids (PG/CL). Samples included 1 µg (PmtS2) or 20 µg of protein (PmtR1 and PmtS1), 50 µg of SAM, 50 µg of the respective lipid substrate (PE, MMPE, or DMPE), and, optionally, 10 µg of anionic lipid (PG or CL). All *in vitro* assays were performed at 30°C for 16 h, after which the lipids were extracted, separated by TLC, and stained using CuSO_4_. The position of commercially available C18:1 phospholipids is indicated. Arrows highlight formed products.

As an initial purification step, all Pmts were subjected to Ni-affinity chromatography. Only small amounts of PmtS1 were recovered due to low overproduction (Fig. 3A), and most of the soluble protein was lost in the flow-through of the Ni-IDA column (data not shown). Consequently, further purification steps were not pursued for this protein (Fig. 4A, left panel). Prior to the activity assay, the elution fraction from the Ni-IDA column underwent buffer exchange to remove imidazole. Following purification and buffer exchange, small amounts of MMPE were associated with PmtS1, as demonstrated by the sample without added lipids (Fig. 4A, right panel, first lane). The addition of PE resulted in slightly increased MMPE levels. As demonstrated previously, the anionic lipids phosphatidylglycerol (PG) or cardiolipin (CL) stimulate Pmt binding to the membrane, thereby enhancing their activity (31, 45). Similarly, the activity of PmtS1 toward PE was enhanced by both PG and CL, as evidenced by increased MMPE levels, although the effect of CL was less pronounced. PmtS1 did not methylate MMPE or DMPE, neither in the presence nor absence of anionic lipids. Considering the significant accumulation of MMPE upon *pmtS1* expression in *E. coli* (Fig. 3B) and the conversion of only PE to MMPE by the purified protein (Fig. 4A), we conclude that PmtS1 primarily catalyzes the first methylation step from PE to MMPE.

During the purification of PmtS2, most of the protein was lost after cell lysis, likely due to inclusion body formation (data not shown). However, enough PmtS2 was eluted after affinity chromatography, which was then concentrated and further purified using size exclusion chromatography (SEC), resulting in PmtS2 and one additional protein of unknown identity (Fig. 4B). PmtS2 was able to methylate all tested substrates *in vitro*. PE was methylated to MMPE and small amounts of DMPE and PC, especially in the presence of PG. PmtS2 also catalyzed the conversion of MMPE to DMPE and PC in the presence of PG. Lastly, DMPE was methylated to PC, and this activity was stimulated by PG. Based on these results, we conclude that PmtS2 catalyzes all three methylation steps from PE to PC.

PmtR1, the sole R-type Pmt in *R. leguminosarum*, was successfully purified in substantial amounts and with good purity after SEC (Fig. 4C). *In vitro* assays revealed that PmtR1 does not utilize PE as a substrate, irrespective of the presence or absence of anionic lipids. When MMPE was employed as a substrate, only barely detectable amounts of PC were synthesized in the absence of anionic lipids or in the presence of CL. However, this activity was stimulated by PG, resulting in increased PC accumulation. Interestingly, DMPE proved to be the most efficiently used substrate for PmtR1. Large amounts of PC were produced under all tested conditions. In conclusion, PmtR1 does not efficiently catalyze the initial methylation step from PE to MMPE. Instead, it catalyzes the reaction from MMPE to DMPE and, most efficiently, from DMPE to PC. This substrate specificity explains the low activity observed when this protein was produced in *E. coli* (Fig. 3), as *E. coli* membranes naturally lack the necessary methylated substrates MMPE or DMPE.

### Abundance of PC biosynthesis enzymes in *R. leguminosarum*

The heterologous production and *in vitro* assays of the enzymes putatively involved in PC biosynthesis in *R. leguminosarum* have provided insights into their activity and substrate preferences. It appears that multiple enzymes participate in PC biosynthesis in this plant symbiont. This raises the question of whether all these enzymes are produced and, if so, whether they are active simultaneously in *R. leguminosarum*.

**Fig 5:**
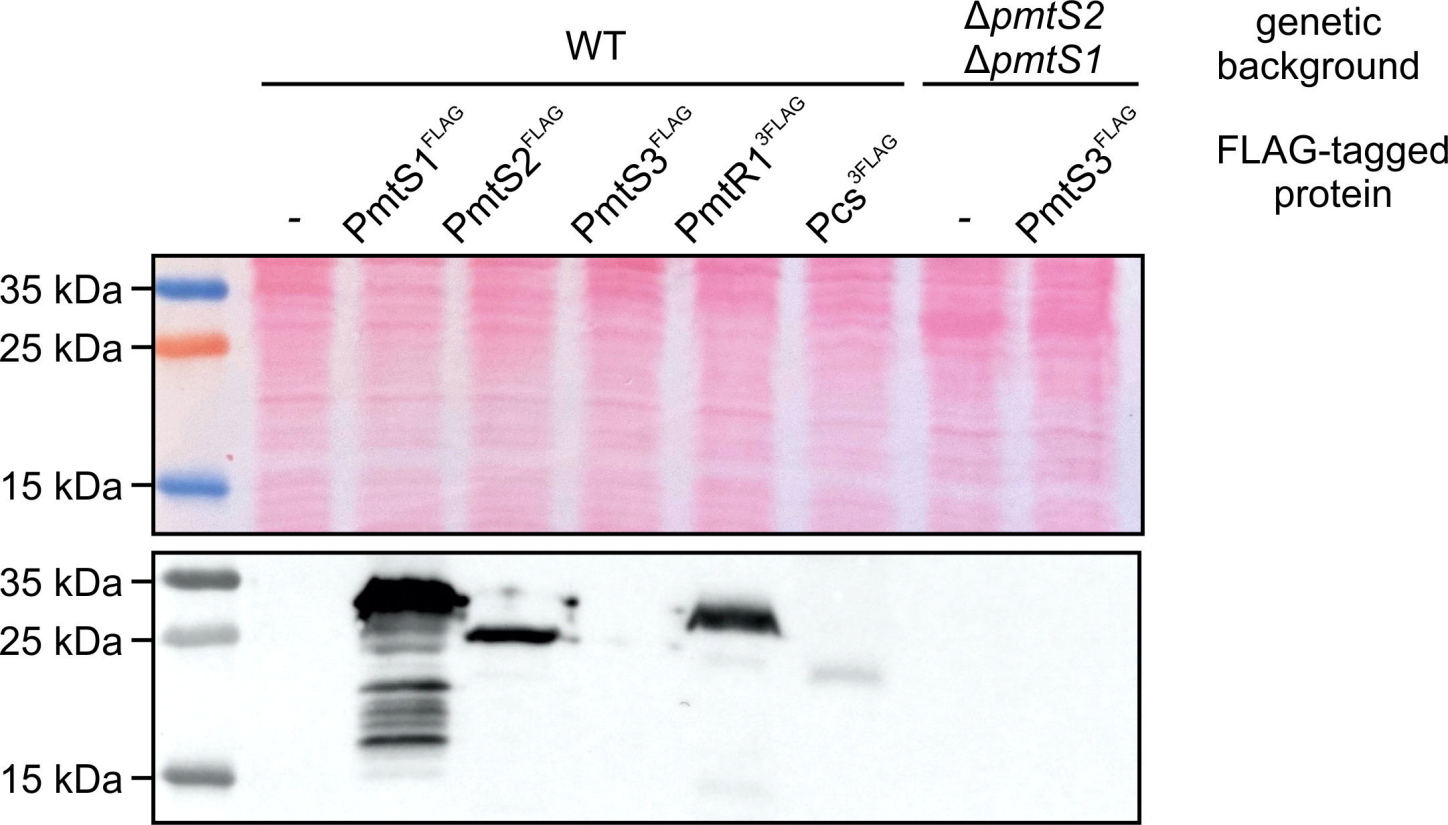
Production of FLAG-tagged PC biosynthesis enzymes from their authentic gene loci in *R. leguminosarum*. The *R. leguminosarum* FLAG-reporter strains were grown in YEM medium for 3 days at 30°C. Subsequently, cells were harvested, and proteins were analyzed via SDS-PAGE and Western blotting. The tagged proteins were detected using FLAG-specific antibodies. The upper panel displays the Ponceau S-stained membrane before blotting as a loading control.

To address the question, we created reporter strains with C-terminally FLAG-tagged enzymes via integration into their authentic gene loci. The reporter strains were cultivated in yeast extract mannitol medium (YEM), and protein amounts were determined by SDS-PAGE and Western blotting using FLAG-specific antibodies. All Pmt proteins, except PmtS3^FLAG^, were detected (Fig. 5). Since PmtS3 was not detectable in the wild type under the tested conditions, we also explored whether it might be produced in the absence of *pmtS2* and *pmtS1* to compensate for their absence. However, PmtS3^FLAG^ was not observed in the double mutant ∆*pmtS2*∆*pmtS1*. Additionally, Pcs^FLAG^ was detected, albeit in lower quantities compared to the Pmts. This observation suggests that both pathways, Pmt-mediated methylation and the PC synthase, operate simultaneously in *R. leguminosarum*.

### PC biosynthesis enzymes in *R. leguminosarum pmt* and *pcs* mutants

**Fig 6:**
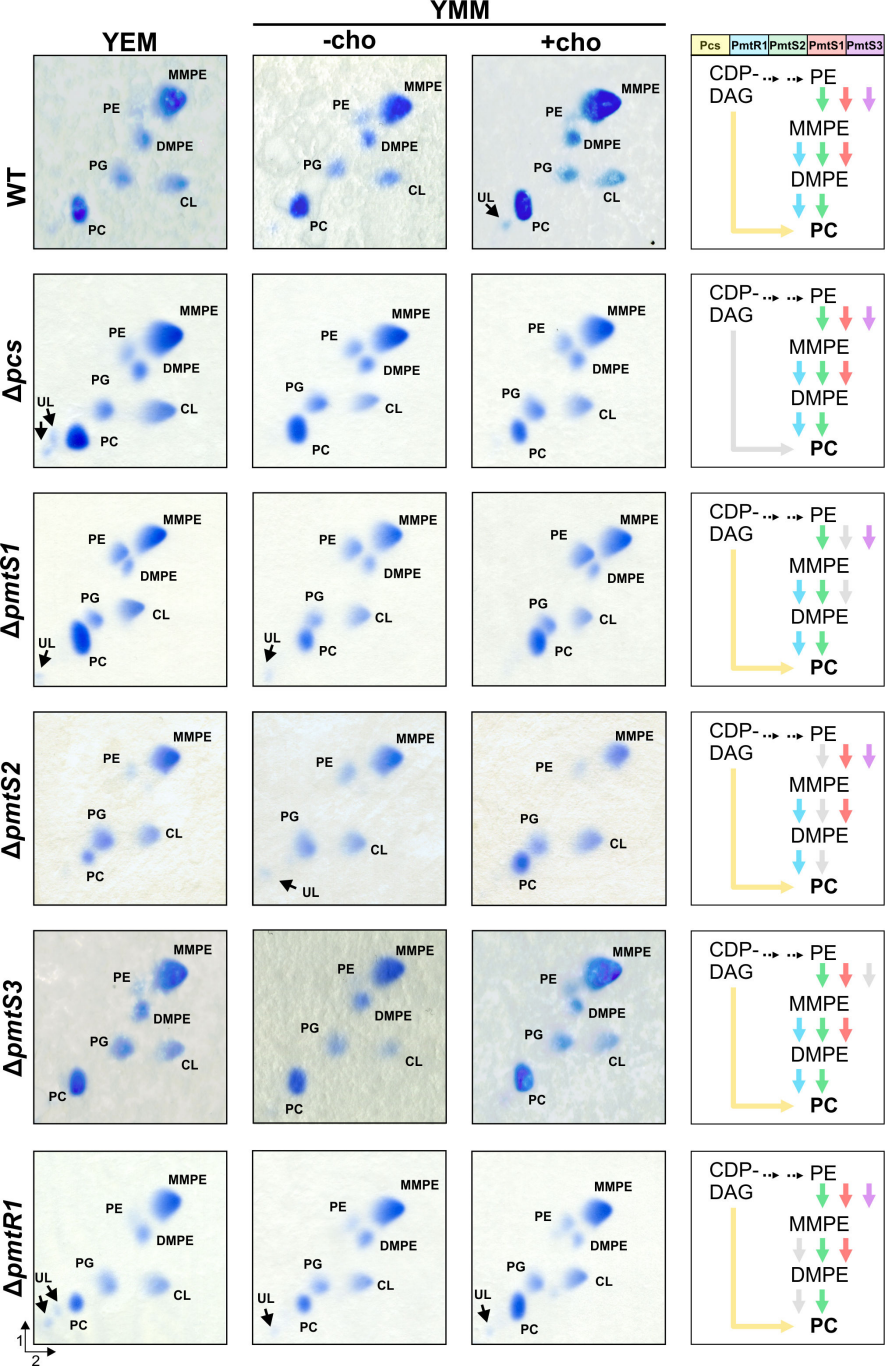
Lipid composition of *R. leguminosarum* wild type and single deletion mutants. All strains were grown in complex medium (YEM) or minimal medium (YMM) in the absence (−cho) or presence (+cho) of choline. After 3–4 days, cultures were harvested, and lipids were extracted and separated by 2D TLC. Phospholipids were visualized using molybdenum blue reagent. The direction of the first and second separation dimension is indicated. The right column gives an overview over the PC biosynthesis pathways potentially active (indicated by colored arrows) in the respective strain, based on the enzyme activities in *E. coli* and *in vitro*. UL, unknown lipid.

**Fig 7:**
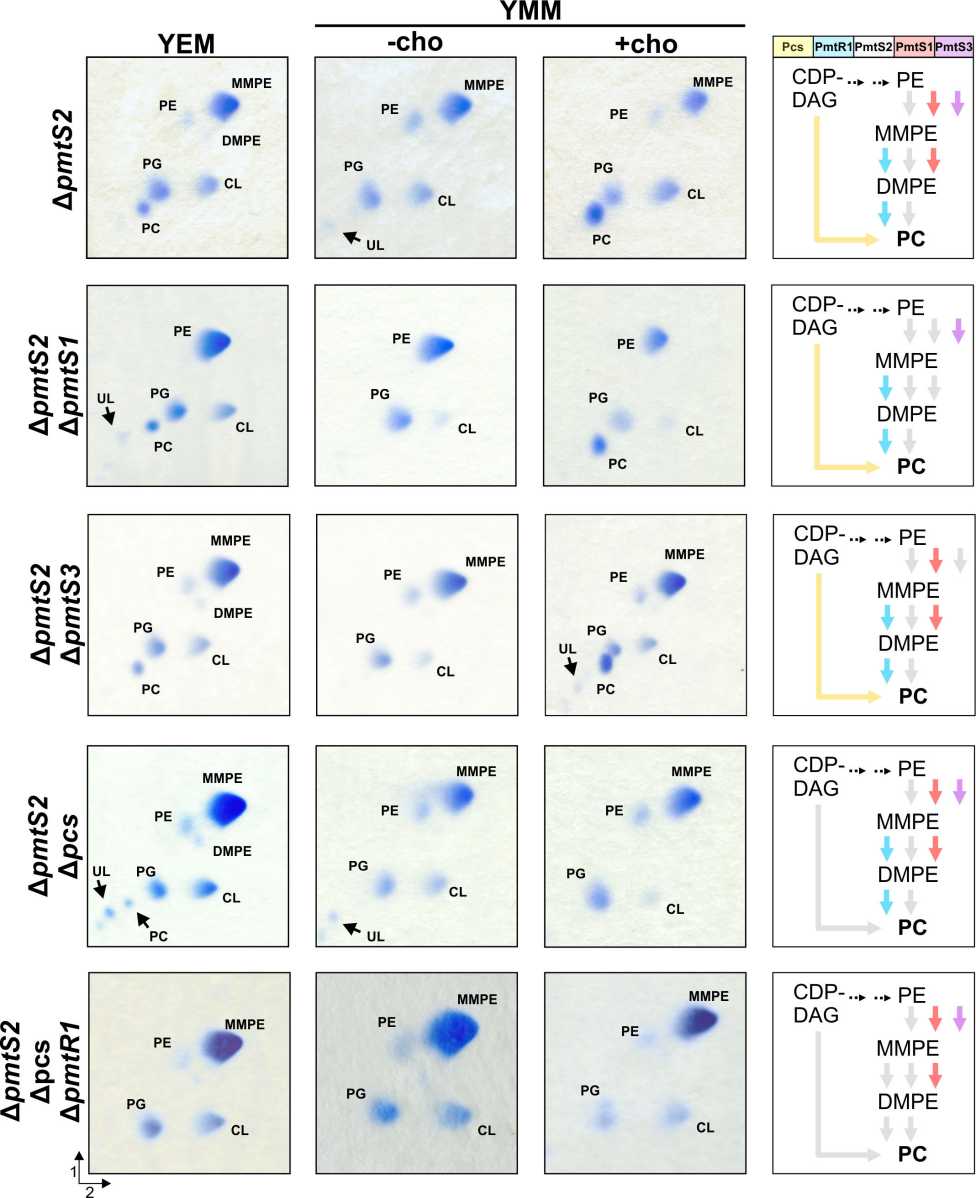
Lipid composition of *R. leguminosarum* double or triple deletion mutants. For comparison, the Δ*pmtS2* panel from Fig. 6 is included. All strains were grown in complex medium (YEM) or minimal medium (YMM) in the absence (−cho) or presence (+cho) of choline. After 3–4 days, cultures were harvested, and lipids were extracted and separated via 2D TLC. Phospholipids were visualized using molybdenum blue reagent. The direction of the first and second separation dimension is indicated. The right column gives an overview over the PC biosynthesis pathways potentially active (indicated by colored arrows) in the respective strain, based on the enzyme activities in *E. coli* and *in vitro*.

After confirming the presence of Pmt and Pcs proteins in their native host, we assessed the contribution of these enzymes to PC biosynthesis by constructing deletion mutants and analyzing whether the absence of a specific gene influenced the membrane phospholipid composition of *R. leguminosarum*. All strains were cultivated in both complex medium and minimal medium, the latter with and without choline supplementation. Choline was included since the Pcs pathway relies on exogenous choline as a substrate. Subsequently, the phospholipid profile of the mutants was analyzed by two-dimensional (2D) TLC (Fig. 6 and 7), and the relative amounts of phospholipids were quantified through pixel counting (Tables S3 and S4).

The composition of the *R. leguminosarum* bv. *trifolii* ATCC14479 membrane remained similar across all three growth media conditions, with the predominant phospholipids being MMPE (41%–44%) and PC (24%–27%). Lower quantities of DMPE (8%–10%), PG (7%–10%), and CL (10%–11%) were observed, while PE was detected in trace amounts (3%–4%) (Fig. 6; Table S3). Supplementation of choline to Y-minimal medium (YMM) did not significantly increase PC levels. This observation suggests that the PC synthase pathway may not be the primary route for PC biosynthesis in *R. leguminosarum*. This hypothesis is supported by the low protein level of Pcs^FLAG^ detected within the wild type (Fig. 5) and the finding that deletion of *pcs* did not lead to a drastic decrease in the relative PC amount under all tested growth conditions (Fig. 6; Table S3).

Experiments conducted in the heterologous *E. coli* system and with purified enzyme revealed that PmtS1 primarily catalyzes the methylation of PE to MMPE (Fig. 3 and 4). These findings are supported by the approximately 30% decrease in the relative amounts of MMPE, accompanied by an increase in PE, observed in the *pmtS1* deletion mutant under all conditions (Fig. 6; Table S3). However, MMPE remained the most abundant phospholipid in this mutant, suggesting the presence of (an)other MMPE-producing enzyme(s).

Deletion of *pmtS2* had the most severe impact on the phospholipid composition among the single-gene deletion mutants (Fig. 6; Table S3). When cultivated in complex YEM medium, there was a substantial decrease in the relative amount of PC (from 24% to 11%) and DMPE (from 10% to 1%), which correlates with the activity of the protein *in vitro* and in *E. coli* (Fig. 3 and 4). In parallel, the amount of PG increased from 10% to 24%. MMPE (45%) remained the major phospholipid in the *pmtS2* mutant, likely due to the function of PmtS1. In YMM minimal medium without a choline source, rendering the PC synthase pathway inactive, PC was completely absent in the *pmtS2* mutant (Fig. 6). This effect was reversed upon the supplementation of choline, leading to the production of large amounts of PC (31% of total lipids). This choline dependency clearly indicated that Pcs becomes the dominant PC-producing enzyme in the absence of PmtS2 provided that choline is available. Neither Pcs nor any other Pmt could compensate for the lack of DMPE in the *pmtS2* mutant under all conditions, suggesting that PmtS2 is the primary producer of DMPE in *R. leguminosarum*.

In line with the absence of PmtS3^FLAG^ in the wild-type *R. leguminosarum* strain (Fig. 5), deletion of the *pmtS3* gene had no discernible effect on phospholipid composition (Fig. 6; Table S3). Therefore, PmtS3 might not play an active role in PC biosynthesis in *R. leguminosarum* under the tested conditions.

Dependent on the growth medium, the deletion of the *pmtR1* gene resulted in a 20%–40% decrease of the relative PC amount compared to the wild type, supporting an active role of PmtR1 in PC biosynthesis in *R. leguminosarum*. The PC level was the lowest in minimal medium without choline and could be restored to wild-type levels by supplementation with choline, likely due to Pcs activity.

The deletion of individual genes confirmed the functional role of several of the encoded PC biosynthesis enzymes in *R. leguminosarum*. However, due to functional redundancies, the absence of single enzymes might be compensated by isoenzymes. To further elucidate the *in vivo* activities of these enzymes, four double mutants and a triple deletion mutant were created using the Δ*pmtS2* mutant as the genetic background. As with the single deletion mutants, the phospholipid composition of these mutants grown in complex medium and minimal medium was profiled by 2D TLC (Fig. 7; Table S4).

The presence of normal MMPE amounts in the *pmtS2* mutant (Fig. 6; Table S4) raised the question of whether PmtS1 or PmtS3, or both, produced the large quantities of MMPE. The phospholipid profile of the ∆*pmtS2*∆*pmtS1* and ∆*pmtS2*∆*pmtS3* mutants clearly demonstrated that PmtS1 was responsible for this activity. While MMPE was absent under all conditions tested, large amounts of PE (45%–66%) accumulated in the ∆*pmtS2*∆*pmtS1* mutant (Fig. 7; Table S4). Due to similar retention behaviors of PE and MMPE in 2D TLC, the unambiguous identification of one of them can be challenging when the other is missing. To circumvent this, the identity of the PE spot was verified by one-dimensional (1D) TLC using a solvent system that separates the two spots more clearly (Fig. S1A). Additionally, ninhydrin staining of the lipids separated by 1D TLC confirmed the identity of PE, as MMPE and PE are stained in different colors due to their difference in free amino groups (Fig. S1B). We conclude that PmtS3 does not play an active role in the methylation pathway in *R. leguminosarum*, even though the recombinant enzyme is able to produce MMPE in *E. coli* (Fig. 3B). This discrepancy can probably be explained by the absence of the enzyme in both the *R. leguminosarum* wild type and Δ*pmtS2*Δ*pmtS1* mutant, as indicated by the PmtS3^FLAG^ construct (Fig. 5).

The Δ*pmtS2*Δ*pcs* mutant contained traces of PC in YEM medium, likely due to PmtR1 activity. This was supported by a triple deletion mutant lacking *pmtS2, pcs,* and *pmtR1*, where PC was completely absent in all media tested (Fig. 7; Table S4). The membrane of the Δ*pmtS2*Δ*pcs* and the Δ*pmtS2*Δ*pcs*Δ*pmtR1* mutant was mostly composed of MMPE (72%), which can only derive from PmtS1 activity (Fig. 7; Table S4).

### Alternative substrates for PC biosynthesis in *R. leguminosarum*

**Fig 8:**
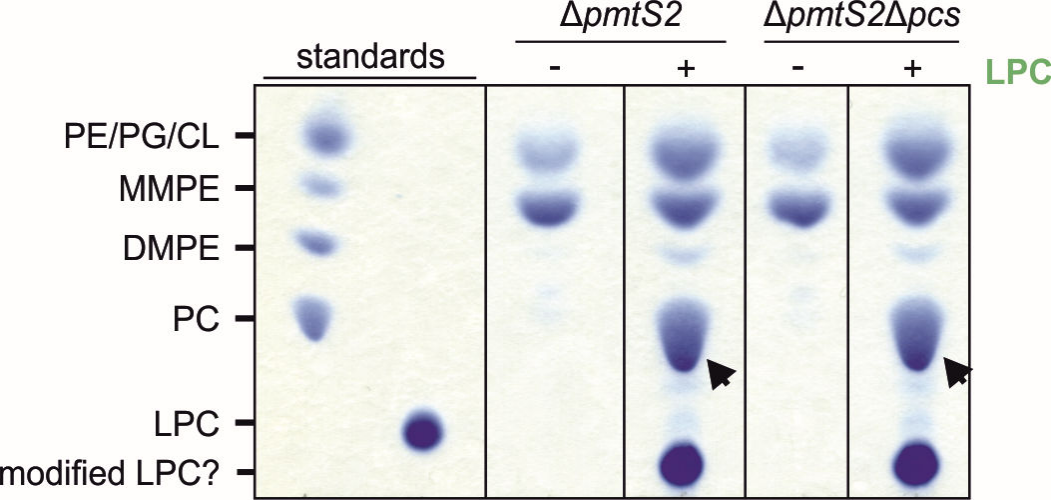
*R. leguminosarum* utilizes LPC as an alternative substrate for PC production. The *R. leguminosarum* Δ*pmtS2* and Δ*pmtS2*Δ*pcs* mutants were grown in YMM minimal medium in the absence (−) or presence of 0.05 mM lyso-PC as Triton X-100 mixed micelles (0.05%). All lipids were extracted, separated by TLC, and visualized using molybdenum blue reagent. The position of commercially available C18:1 phospholipids is indicated. Arrows highlight formed products.

After deciphering the complexity of the Pmt pathway, we wondered whether *R. leguminosarum* can produce PC utilizing alternative substrate sources (Fig. S2; Fig. 8). The Kennedy pathway, a common route for PC formation in eukaryotes, involves the condensation of CDP-choline with DAG catalyzed by cholinephosphotransferases. In bacteria, this pathway has only been identified in *Treponema denticola* (46), and contrasts the conventional bacterial pathway mediated by Pcs, which utilizes choline and CDP-DAG to produce PC.

We investigated whether *R. leguminosarum* can utilize CDP-choline for PC synthesis and used the Δ*pmtS2* mutant to minimize the PC background. The Δ*pmtS2* mutant was cultivated with and without CDP-choline prior to lipid analysis. The addition of CDP-choline resulted in the synthesis of PC in the Δ*pmtS2* mutant, illustrating the organism’s capacity to utilize externally supplied CDP-choline for PC production (Fig. S2A, left panel). To investigate whether PC synthesis from CDP-choline relies on Pcs or requires other enzymes, we used the Δ*pmtS2*Δ*pcs* mutant. Cultivation of the Δ*pmtS2*Δ*pcs* mutant with CDP-choline did not yield any PC production, demonstrating that Pcs is a prerequisite for PC production from CDP-choline. The contribution of Pcs in CDP-choline-dependent PC formation was confirmed with *E. coli* BL21 (DE3) cells expressing *pcs* (Fig. S2A).

In the acylation pathway, described in only a few bacteria so far, glycerophosphocholine is first acylated to lyso-PC and then further to PC using acyl-CoA as a substrate (30, 43, 47). Feeding GPC to *R. leguminosarum* resulted in PC production; however, as described for CDP-choline, this occurred only in a Pcs-dependent manner (Fig. S2B). We hypothesize that choline is liberated from both GPC and CDP-choline, either externally or internally, subsequently acting as a substrate for the Pcs pathway for PC production.

We then asked whether *R. leguminosarum* can catalyze the acylation from LPC to PC and supplemented the lyso-lipid to the growth medium. In both the Δ*pmtS2* and Δ*pmtS2*Δ*pcs* mutants, large amounts of PC were produced when LPC was present (Fig. 8). These findings suggest that *R. leguminosarum* can generate PC from LPC independently of Pcs, potentially through the activity of one or more presently unidentified acyltransferase(s). It is worth noting that the supplemented LPC undergoes some modification within the cells, as evidenced by a distinct migration pattern in the lipids extracted from the bacterium compared to the added C18:1 LPC in the medium.

### Symbiotic efficiency of *R. leguminosarum* deletion mutants

It has been reported that nodules formed by PC-deficient *B. diazoefficiens* or *S. meliloti* strains are unproductive in nitrogen fixation (17, 20, 21, 25). We sought to determine whether PC deficiency in *R. leguminosarum* affects symbiosis with red clover. If so, our aim was to decipher which PC biosynthesis pathway is responsible for this phenotype.

**Fig 9:**
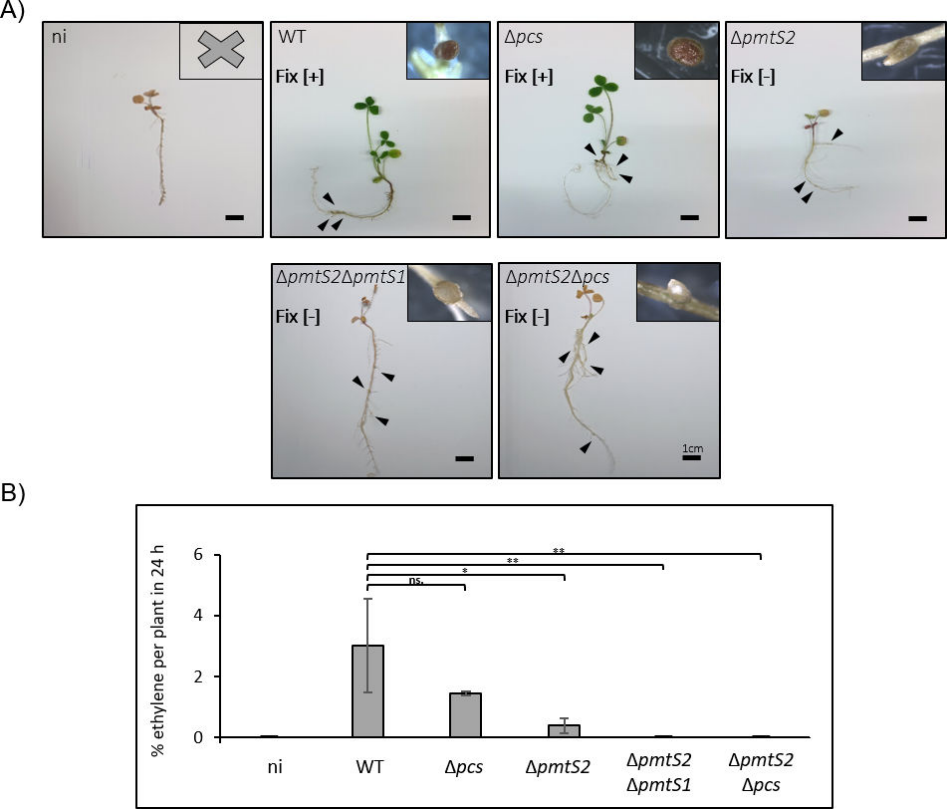
Appearance and acetylene reduction activity of red clover inoculated with *R. leguminosarum* wild type (WT) and mutant strains. (**A**) Red clover seedlings were grown in Fahraeus agar and inoculated with *R. leguminosarum*. Plants were incubated at controlled conditions [21°C for 16 h (day) and 17°C for 8 h (night)] and harvested after 5 weeks. One representative plant is shown. Arrow heads indicate developed nodules depicted while cut open to the upper right of the respective plant. (**B**) Nitrogen fixation estimated by the ARA. The initial concentration of acetylene in the sample was set to 100%. After 24 h, the amount of conversion product (ethylene) was determined as a metric for fixed acetylene (acetylene to ethylene conversion ratio of 1:1). ni, not inoculated plant. The visual appeal of the plant, the color of the cut nodules (red, with leghemoglobin; white, without), and the percentage of acetylene fixation per plant were used to assess successful (Fix [+]) or failed nitrogen fixation in symbiosis with clover plants (Fix [−]). Significance (*t*-test): *P*-values * <0.05, ** <0.03. ns: not significant.

We inoculated red clover seedlings with the *R. leguminosarum* wild type and selected mutants with varying levels of PC production (Δ*pcs,* Δ*pmtS2,* Δ*pmtS2*Δ*pmtS1,* Δ*pmtS2*Δ*pcs*; Fig. 6 and 7). The symbiotic efficiency was measured based on the number of formed nodules, the dry weight of the plant foliage, and the results from the acetylene reduction assay (ARA). As the growth medium of the plants did not contain any nitrogen source, plants were dependent on the nitrogen fixation activity of *R. leguminosarum* in the root nodules. After 5 weeks, nodules were found on all plants except for the uninoculated controls, as expected (Table 1; Fig. 9A). The average number of nodules did not differ significantly between the PC biosynthesis mutants and the wild type, indicating that the presence of PC is not essential for nodule formation by *R. leguminosarum*.

**TABLE 1:** Number of nodules and foliage weight of clover plants inoculated with *R. leguminosarum* wild type and deletion mutants^*a*^

	*R. leguminosarum* inoculant	No. of nodules	Foliage weight (mg)
Red clover (*T. pratense*)	Not inoculated	–^*b*^	7.9 (±2.9)
	Wild type	5.9 (±1.5)	18.2 (±14.5)
	Δ*pcs*	5.0 (±2.3)	22.1 (±13.9)
	Δ*pmtS2*	7.7 (±2.9)	7.9 (±2.1)
	Δ*pmtS2*Δ*pmtS1*	5.9 (±3.1)	8.8 (±4.5)
	Δ*pmtS2*Δ*pcs*	3.2 (±2.1)	9.7 (±3.1)

^*a*^ Values represent the average and standard deviation (±) from 10 plants and inoculant strain.

^*b*^ –, zero.

The visual inspection of the inoculated plants revealed that those inoculated with the *R. leguminosarum* wild type had a healthy, green appearance (Fig. 9A) and an average foliage weight of approximately 18 mg (Table 1). Their nodules exhibited a characteristic red color, indicative of the presence of leghemoglobin, and displayed acetylene reduction activity (Fig. 9A and B). Plants infected with the *pcs* mutant showed comparable foliage appearance, weight, and red-colored nodules to those infected with the wild type. However, the acetylene fixation activity was slightly reduced. Similar results were made for mutants lacking *pmtR1, pmtS1,* and *pmtS3* (Fig. S3).

Conversely, plants inoculated with mutant strains lacking the *pmtS2* gene exhibited stunted growth and looked pale with reddish leaves reminiscent of the uninoculated negative controls. These plants displayed reduced foliage weight, and the nodules appeared white lacking the characteristic coloration of leghemoglobin. The nodules were deficient in nitrogen fixation as indicated by the reduced acetylene reduction activity (Fig. 9A and B). The marked reduction or absence of PC in the *pmtS2* mutants (Fig. 6 and 7) suggests that PmtS2 is the PC biosynthesis enzyme in *R. leguminosarum* required for providing adequate PC levels to sustain efficient symbiosis with clover.

## DISCUSSION

Nitrogen-fixing plant symbionts, such as *R. leguminosarum,* are of significant interest in agriculture due to the frequent limitation of crop yields by nitrogen availability (48). Therefore, a comprehensive understanding of the underlying mechanisms governing this symbiosis is important. One crucial aspect influencing the interaction between a bacterium and its eukaryotic host often is the presence of PC in the bacterial membrane (17, 18). Previous research has demonstrated the severe impact of PC deficiency on the nitrogen fixation activity of *B. diazoefficiens* and *S. meliloti* (20, 21, 25). Hence, it was reasonable to assume that the symbiosis of *R. leguminosarum* might also be influenced by the presence of PC. It had been suggested that the clover symbiont possesses the capability to produce PC through the PC synthase and methylation pathways (36). However, the functionality of specific PC biosynthesis enzymes and their contribution to the outcome of plant-microbe interaction remained elusive. In this study, we elucidated how *R. leguminosarum* synthesizes PC and demonstrated the importance of this phospholipid for the nitrogen-fixing symbiosis with clover.

### One out of four phospholipid *N*-methyltransferases is crucial for nitrogen fixation

**Fig 10:**
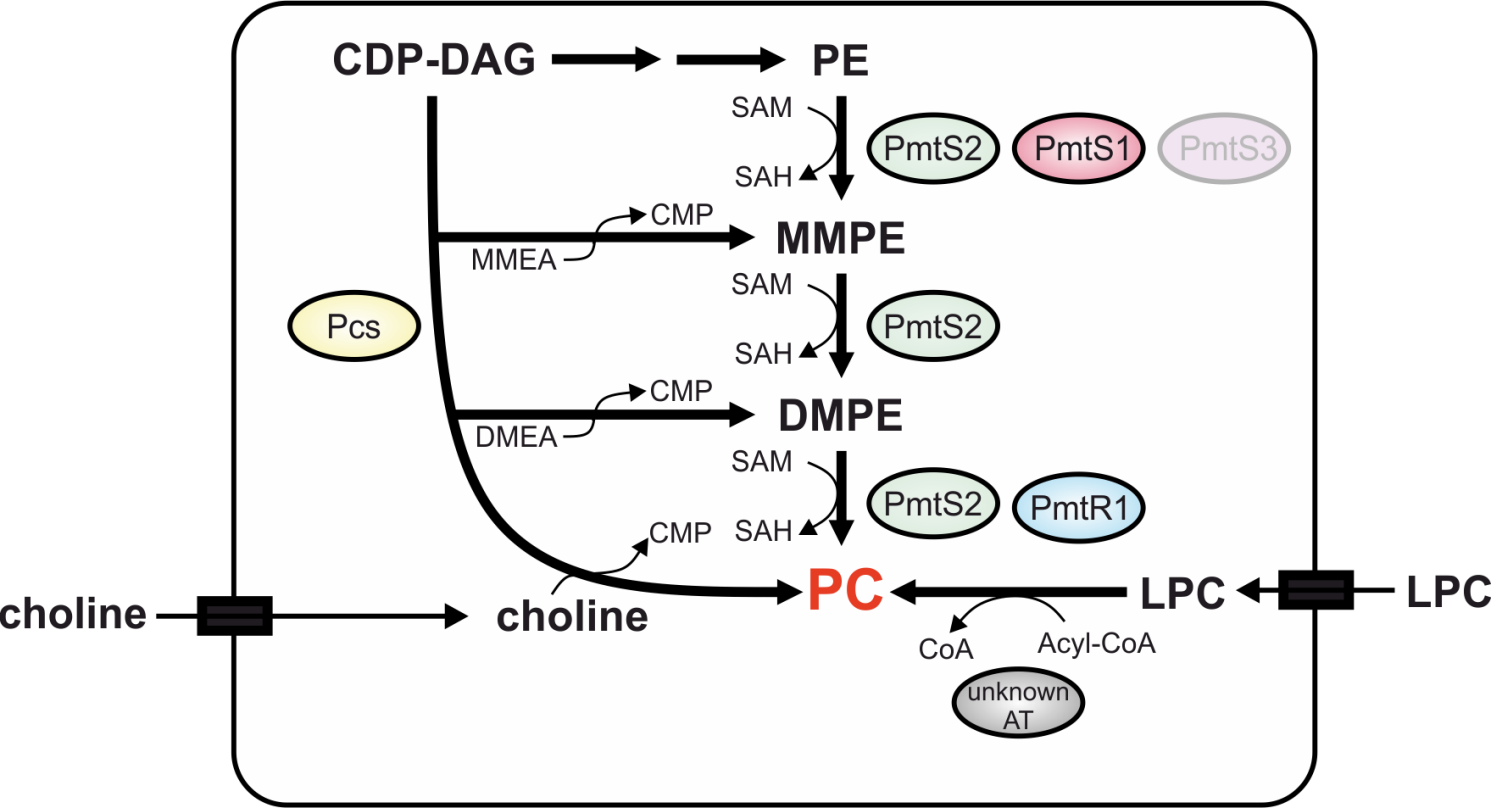
Overview of PC biosynthesis pathways in *R. leguminosarum*. The bacterium can synthesize PC via the methylation pathway, for which three out of four Pmts (PmtS2, PmtS1, and PmtR1) were shown to be active in this study. PmtS3, which was not detected under laboratory conditions in *R. leguminosarum* but was active in *E. coli,* is grayed out. In addition, the Pcs enzyme produces PC by condensation of CDP-DAG with choline, which can be acquired from the environment. Moreover, *R. leguminosarum* forms PC in the presence of LPC by the action of one or more yet unidentified acyltransferase(s) (AT). CMP, cytidine monophosphate; CoA, coenzyme A; SAM, *S*-adenosyl methionine; SAH, *S*-adenosyl homocysteine.

PC biosynthesis in *R. leguminosarum* is remarkably complex and flexible (Fig. 10). Among the array of pathways available to produce PC from both external and internal substrates, the methylation pathway emerged as the predominant pathway necessary for the establishment of productive root nodules. Out of the four Pmt candidates, PmtS2 turned out to be the key enzyme responsible for carrying out all three methylation reactions from PE to PC, thus rendering it indispensable for symbiotic nitrogen fixation. Now the question arises why *R. leguminosarum* possesses multiple Pmt enzymes, given that PmtS2 alone is capable of synthesizing PC, at least under the conditions tested. One explanation might be that multiple Pmt enzymes offer redundancy and robustness to the biosynthetic pathway. In situations where one enzyme lacks substrate or is inactivated due the mutations or inhibitors, redundancy guarantees that the organism can still generate PC or other methylated PE derivatives through alternative Pmts. MMPE, the most abundant phospholipid with largely unexplored functions in *R. leguminosarum*, must also be considered. Some Pmts, especially PmtS1, and probably PmtS3 under certain conditions, might play a pivotal role in maintaining the exceedingly high MMPE levels.

In comparison, PC biosynthesis in *S. meliloti* appears straightforward. The organism encodes one PmtA enzyme catalyzing all three methylation steps and one Pcs enzyme. The double-knockout strain exhibits reduced growth (49), loss of motility, succinoglycan overproduction, and an inability to form nitrogen-fixing root nodules on alfalfa (25). In contrast, PC biosynthesis in *R. leguminosarum* resembles an equally complex situation in *B. diazoefficiens*. In *B. diazoefficiens,* the methylation pathway operates in a two-step process: the S-type PmtA methylates PE to MMPE and DMPE, followed by the R-type PmtX1 utilizing MMPE and DMPE as substrates for subsequent methylation to PC (20). The *pmtA* mutant was strongly impaired in nitrogen fixation, while a *pmtX1* mutant could not be constructed, underscoring the critical role of PC in bacterial physiology (21, 50). Two additional Pmt candidates from *B. diazoefficiens* were found to be active in *E. coli* with different substrate specificities. While the corresponding genes were not expressed in *B. diazoefficiens* under standard laboratory conditions, one of them was upregulated in the *pmtA* mutant suggesting potential compensation for the absence of PmtA (50). Similarly, *R. leguminosarum* harbors four Pmts with distinct substrate and product specificities (Fig. 10). Through successful purification and biochemical characterization of three of these enzymes, we obtained a coherent picture of the PC biosynthesis pathways corroborated by multiple lines of evidence both *in vitro* and *in vivo* (expression in *E. coli*; deletion mutants and FLAG-tag strains in *R. leguminosarum*).

PmtS3 is a notable exception for several reasons. The protein was undetectable in both the *R. leguminosarum* wild type and Δ*pmtS2*Δ*pmtS1* mutant. Unlike all other genes involved in PC biosynthesis, *pmtS3* is located on one of the four plasmids. The genome of *R. leguminosarum* strains typically consists of a chromosome and a variable number of plasmids (Table S5). The core chromosome is highly conserved, containing most essential genes, while plasmid-encoded genes are often regarded as accessory and adaptive (51, 52). The extrachromosomal DNA exhibits high variability between strains, e.g., to facilitate adaptation to specific symbiotic partners. Consequently, certain genes are only present in a subset of *R. leguminosarum* strains. This is evident with *pmtS3,* which is absent from 6 out of 21 fully sequenced genomes available in the NCBI database (Table S5). Remarkably, all other chromosomally encoded *pmt* genes, as well as *pcs*, are conserved across all genomes. The absence of *pmtS3* in one-third of the strains investigated underscores its minor importance for *R. leguminosarum*. It is possible that an evolutionary ancestor originally encoded only three Pmts and that the fourth gene was later acquired by certain strains. Alternatively, it could be hypothesized that an evolutionary ancestor encoded four Pmts and that *pmtS3* was lost from the plasmid in some strains through recombination events. Little selection pressure due to the functional redundancy of PmtS3 with PmtS1, which is encoded on the chromosome, likely contributed to this loss.

### Multiple ways to produce PC

The methylation pathway in *R. leguminosarum* is complemented by additional PC biosynthesis options (Fig. 10). Beyond the canonical choline condensation process, the Pcs enzyme was able to accept MMEA and DMAE as substrates, yielding MMPE and DMPE, respectively. This broad substrate spectrum seems to be a conserved feature of Pcs enzymes (34, 42–44). Presumably, this substrate promiscuity equips the bacterium with significant adaptability to synthesize all three methylated PE derivatives via Pcs and/or Pmt activity, depending on the availability of precursors from the environment. Bacteria generally cannot produce choline *de novo* and thus rely on the uptake of exogenous choline from the environment (26, 27, 36, 53). To attract symbiotic bacteria, plants are believed to exude choline into the environment (54). When choline was available, Pcs effectively replenished PC in the *R. leguminsarum pmtS2* mutant to levels comparable to the wild type (increasing from 0% without choline to 31% with choline; Table S3). However, it appears that the choline levels in the plant infection assay were inadequate for PC production, as evidenced by the severely compromised nitrogen fixation activity in the *pmtS2* mutant but not in the *pcs* mutant.

*R. leguminosarum* produced PC when CDP-choline was supplemented to the growth medium (Fig. 10). Notably, previous *in vitro* assays with *A. tumefaciens* Pcs revealed that the enzyme did not utilize CDP-choline and DAG as substrates for PC production (data now shown). This suggests that CDP-choline in *R. leguminosarum* was metabolized within the cell to choline, which was then condensed with CDP-DAG to form PC through the conventional Pcs activity (Fig. 10).

Pcs-dependent PC production was also observed in the presence of GPC (Fig. S2B). While certain bacteria, such as *X. campestris,* can acylate GPC to LPC, which is then utilized for PC production, *R. leguminosarum* exclusively generated PC from LPC in a Pcs-dependent manner. Analogous to CDP-choline, it is likely that the bacterium derives choline from GPC.

When LPC was provided, *R. leguminosarum* was able to acylate it into PC (Fig. 10). In contrast to the CDP-choline and GPC-dependent PC formation, this activity was found to be independent of Pcs, which strongly suggests the existence of one or more acyltransferase(s) capable of adding an acyl chain to the lysolipid. While *R. leguminosarum* was able to produce large amounts of PC from LPC under laboratory conditions, it remains uncertain whether and how much LPC the bacterium encounters in its natural habitats. A recent study on lipids in soil organic matter revealed that LPC constitutes approximately 4% of all lipids in soil and around 8% of all lipids in roots (55). However, it is unclear how much of this LPC exists in a free state, available for incorporation and acylation by *R. leguminosarum,* vs being part of the membranes of other organisms present in the soil. Nevertheless, it is conceivable that *R. leguminosarum* may encounter trace amounts of LPC in its environment, leading to the production of minor quantities of PC via the acylation pathway.

### PC is crucial for symbiotic nitrogen fixation

To the best of our knowledge, *R. leguminosarum* is now the third rhizobia member demonstrated to depend on PC for effective symbiosis with host plants (21, 25). Similarly, deficiency in PC within the phytopathogen *A. tumefaciens* abrogated tumor formation on plants (26). Interestingly, the severity of these defects in plant-microbe interactions varies. In the case of the *S. meliloti pmtA/pcs* mutant, the absence of PC completely abolished nodule formation at an early developmental stage (25). Conversely, both the *B. diazoefficiens pmtA* mutant and the *R. leguminosarum pmtS2* mutant, which retained some capacity to produce residual PC, progressed to nodule formation (21). However, these nodules appeared pale and lacked the ability to fix nitrogen. Despite these striking phenotypes, the molecular mechanisms underlying the significance of PC in plant-microbe interactions remain poorly understood.

It has been suggested that signal transduction processes, such as the functionality of the sensor kinase VirA in *A. tumefaciens*, may be disrupted in PC-depleted membranes (56), but this hypothesis requires further investigation. Additional support for the connection between PC and membrane-associated signal transduction processes comes from a recent suppressor screen conducted with the *S. meliloti* PC-deficient mutant (25). This strain exhibits a loss of swimming ability and an overproduction of succinoglycan due to reduced transcripts necessary for flagellum formation and increased transcripts for succinoglycan biosynthesis. Several suppressor mutants from this screen, which regained normal swimming ability and succinoglycan levels, showed alterations in the ExoS sensor kinase. The authors proposed that the physicochemical properties of PC-deficient membranes induce conformational changes in the membrane-embedded sensor protein, leading to premature activation of the ExoS-dependent signaling cascade. Other suppressor mutants from the same screen exhibited various amino acid substitutions in ExoS, the ChvI response regulator, the sigma factor RpoH1, or FabA (57). Intriguingly, the mutations restored the ability to form nodules on alfalfa roots, albeit to varying degrees. However, nodules formed by the suppressor strains appeared white instead of pink and were unable to fix nitrogen, resulting in yellow, stunted host plants.

The precise point at which the blockage between nodule formation and successful nitrogen fixation occurs in PC-deficient rhizobia is currently unknown. It appears that some step after infection thread formation is compromised. Despite adjustments in membrane composition by PC-deficient *S. meliloti fabA* supressor mutants, characterized by lower levels of unsaturated fatty acids and higher levels of saturated and shorter chain fatty acids to be compensated for the lack of PC, the tri-methylated phospholipid seems indispensable for later stages of nodule development and efficient nitrogen fixation activity (57). Lipidomic and transcriptomic profiling of developing soybean nodules has revealed that active fatty acid and lipid metabolism are essential for nodulation and productive symbiosis. Notably, PC ranks among the most abundant lipids in developing nodules (58). Our study further underscores the importance of PC in symbiotic plant-microbe interactions. Future research is needed to uncover both common and divergent principles in the requirement for PC in the symbioses between *S. meliloti* and alfalfa, *Bradyrhizobium* and soybean, and *R. leguminosarum* and clover.

## MATERIALS AND METHODS

### Material

Phospholipids [1,2-dioleoyl-*sn*-glycero-3-phosphoethanolamine (PE), 1,2-dioleoyl-*sn*-glycero-3-phospho-(1'-rac-glycerol) (PG), 1,2-dioleoyl-*sn*-glycero-3-phosphocholine (PC), 1*'*,3*'*-bis(1,2-dioleoyl-*sn*-glycero-3-phospho)-glycerol (CL), 1-oleoyl-2-hydroxy-*sn*-glycero-3-phosphocholine sodium salt (LPC), 1,2-dioleoyl-*sn*-glycero-3-phosphoethanolamine-*N*-methyl (MMPE), and 1,2-dioleoyl-*sn*-glycero-3-phosphoethanolamine-*N*,*N*-dimethyl (DMPE)] were purchased from Avanti polar lipids. *S*-(5′-adenosyl)-L-methionine p-toluenesulfonate salt (SAM), 2-(methylamino)ethanol (MMEA), 2-(dimethylamino)ethanol (DMEA), cytidine 5′-diphosphocholine sodium salt hydrate (CDP-choline), glycerophosphocholine, and molybdenum blue spray reagent were obtained from Sigma-Aldrich. All other chemicals used were of analytical grade and commercially available.

### Cultivation of bacteria

Bacterial strains used in this study are listed in Table S1. *Escherichia coli* JM83 served as cloning host, while heterologous gene expression was conducted in *E. coli* BL21 (DE3). Biparental mating in *R. leguminosarum* was performed using *E. coli* S17-1 as donor. All *E. coli* strains were grown in Luria-Bertani broth or in M9 minimal medium at 37°C. *R. leguminosarum* bv. *trifolii* ATCC14479 strains were cultivated in either yeast extract mannitol medium (59) or Y-minimal medium (60) at 30°C. For growth on solid surfaces, the respective media were supplemented with 1.5% agar. When required, antibiotics were added at the following concentrations: ampicillin 100 µg/mL, kanamycin 50 µg/mL (*E. coli*) or 25 µg/mL (*R. leguminosarum*), nalidixic acid 10 µg/mL.

### Plasmid construction

Plasmids and oligonucleotides used in this study are listed in Tables S1 and S2, respectively. For heterologous expression, genes were cloned into pET28b or pET24b vectors. The genes were retrieved either via PCR (*pcs, pmtS1,* and *pmtR1* in pET24b) using chromosomal *R. leguminosarum* bv. t*rifolii* ATCC14479 DNA as template or by gene synthesis (*pmtS1, pmtS2, pmtS3,* and *pmtR1* in pET28b). The DNA fragments were integrated into the expression vector using the restriction sites NdeI and SalI (*pmtS1, pmtS2, pmtS3,* and *pmtR1*) or NdeI and XhoI (*pcs*). The expression plasmids were subsequently transformed into *E. coli* BL21 (DE3) competent cells.

To construct *R. leguminosarum* deletion mutants, the pK19mobsacB suicide vector was used. This involved amplifying 400 bp fragments up- and downstream of the targeted genes through PCR using *R. leguminosarum* chromosomal DNA as template (*pmtS1, pmtS2, pmtR1,* and *pcs*) or gene synthesis (*pmtS3*). The up- and downstream fragments of each gene were successively cloned into the pK19mobsacB vector.

Plasmids for FLAG-reporter strain construction were created using a similar approach. Here, 400 bp fragments up- and downstream of the stop codon of the targeted gene were amplified via PCR with *R. leguminosarum* genomic DNA as template. The stop codon in the upstream fragment was removed. The 3xFLAG epitope-coding sequence was amplified using pBO2337 as template (61). The FLAG-epitope fragment was cloned in frame between the up- and downstream fragments into the pK19mobsacB vector.

After verification by sequencing, all generated plasmids were introduced into *E. coli* S17-1 through transformation before being transferred to *R. leguminosarum* via conjugation as described below.

### Construction of *Rhizobium leguminosarum* deletion mutants and FLAG-reporter strains

For the construction of *R. leguminosarum* deletion mutants and FLAG-reporter strains, the respective pk19mobsacB plasmids were introduced into *R. leguminosarum* via biparental mating using *E. coli* S17-1 as donor (62). Simple crossover events were selected on YEM agar supplemented with kanamycin and nalidixic acid (to prevent *E. coli* growth). Colonies resistant to kanamycin and nalidixic acid were grown in YEM medium containing 1% sucrose for 48 h to promote the second homologous recombination event. Cultures were plated on YEM agar containing 10% sucrose, and the gene deletion or FLAG-tag integration of colonies growing on sucrose was confirmed via colony PCR. To confirm the correct genomic location of the gene deletion, the region 500 bp upstream and downstream of the deleted genes was amplified via PCR and subsequently sequenced.

### Production of recombinant proteins and analysis of activity in *E. coli*

*E. coli* BL21 (DE3) cells carrying the pET expression plasmid of interest were grown at 37°C until an optical density at the wavelength of 580 nm (OD_580_) of ~0.6 was reached before inducing gene expression by 0.1 mM isopropyl-*β*-D-thio-galactopyranoside. Cultures were then incubated at 30°C (activity test in *E. coli*) or 18°C/24°C (protein purification). For the activity test of the proteins in *E. coli*, cultures were incubated for 2 or 20 h, while cultures for protein purification were incubated for 24 h (PmtS2 and PmtR1) or 48 h (PmtS1). The cells were then harvested by centrifugation and used for the detection of *in vivo* enzyme activity or for protein purification. For the detection of *in vivo* protein activity in *E. coli*, overexpression cultures were adjusted to an OD_580_ of 3, and 1 mL was harvested by centrifugation. Cell pellets were subsequently resuspended in 100 µL of distilled water, and lipids were extracted and analyzed by thin-layer chromatography as described under “Lipid analysis,” below.

### Protein purification

Purification of Pmt proteins was conducted as described previously (31, 34). Briefly, proteins were purified by a combination of Ni-IDA affinity chromatography (Protino Ni-IDA 2000 packed columns, Macherey-Nagel) and size exclusion chromatography (Superdex 75 Increase 10/300 Gl, GE health care). An exception was made for PmtS1 due to low protein yields after elution from the Ni-IDA column. Instead of further purification by SEC, the buffer was exchanged to 50 mM KH_2_PO_4_ (pH 8.0) using PD-10 desalting columns (GE Healthcare). Eluted proteins were analyzed by sodium dodecyl sulfate polyacrylamide gel electrophoresis and used for *in vitro* activity tests.

### Protein analysis

Total protein extracts or purified proteins were separated on 12.5% SDS gels. Prior to loading, protein concentrations were assessed using the BCA Protein Assay Kit (Thermo Scientific) following the manufacturer’s instructions. Subsequently, samples were diluted in the appropriate volume of Laemmli sample buffer (63) and then incubated at 95°C for 10 min. Following SDS-PAGE, proteins were either stained with Coomassie or transferred onto a nitrocellulose membrane (GE Healthcare) for Western blotting. For the detection of His-tagged proteins, anti-His horseradish peroxidase-coupled antibodies (1:5,000, Qiagen) were utilized. FLAG-tagged proteins were detected by first incubating with a FLAG-specific primary antibody (1:4,000), followed by incubation with an anti-mouse HRP conjugate (1:5,000) as the secondary antibody. Detection was accomplished through chemiluminescence using the FluorChem SP Multiimager (Alpha Innotech).

### *In vitro* Pmt activity assays

The *in vitro* activity of Pmts was assessed as described previously (34). Each reaction mixture (100 µL) contained 1–20 µg of the respective enzyme, 50 µg SAM, 50 µg lipid substrate (PE, MMPE, or DMPE), and, optionally, 10 µg anionic lipid (PG or CL) in 50 mM KH_2_PO_4_ (pH 8.0). Lipids were pre-dissolved in Triton X-100 (0.05%). Activity assays were run at 30°C for 2 or 16 h before they were stopped by the addition of 375 µL methanol/chloroform (2:1). The lipids were extracted and analyzed by TLC as described below.

### Lipid analysis

A total of 100 µL from either *in vitro* activity assays or cell pellets resuspended in distilled water was used for lipid extraction according to reference (64). The extracted lipids were separated by TLC using TLC silica gel 60 plates (Merck). For one-dimensional TLC, *n*-propanol/propionic acid/chloroform/distilled water (3:2:2:1) was used as mobile phase. For two-dimensional TLC, lipids were separated in a first dimension using chloroform/methanol/distilled water (65:25:4). After 1D separation, the silica plates were turned by 90° and run in a second dimension using chloroform/methanol/acetic acid/distilled water (90:15:10:3.5) as mobile phase. For unspecific staining of lipids, the silica plate was treated with copper sulfate [300 mM CuSO_4_ × 5 H_2_O, 8.5% (vol/vol) phosphoric acid] and incubated at 170°C for 8 min. Phospholipid-specific staining was achieved using molybdenum blue reagent [commercially obtained from Sigma-Aldrich or prepared according to reference (65)]. For staining of lipids containing free amino groups, the silica plates were treated with ninhydrin solution [1.5% (wt/vol) ninhydrin and 3% (vol/vol) acetic acid in *n*-butanol] and subsequently incubated at 140°C for 10 min. Lipids were identified by comparison with the retention behavior of commercially available C18:1 phospholipids.

### Plant assays

To analyze the symbiotic interaction of *R. leguminosarum*, red clover (*Trifolium pratense* L. cv. Rozeta) was employed as the host organism. Clover seeds were surface sterilized as described previously (66) and subsequently germinated on 0.8% water agar plates for 3 days. Germinated seedlings were transferred to Fahraeus agar slants (59) in glass tubes closed with cellulose stoppers. The seedlings were grown under controlled conditions [21°C for 16 h (day) and 17°C for 8 h (night)]. After 2 days, the seedlings were inoculated with *R. leguminosarum*. For this, *R. leguminosarum* was grown in YEM medium at 30°C for 2 days. Cultures were then adjusted to an OD_600_ of 0.2, and the roots of each plant were covered with 100 µL of the culture. After 40 days of incubation at the conditions stated above, root nodules were counted, and the dry weight of the foliage was determined (after drying for 48 h at 60°C). Furthermore, nodules were cut open, and the presence of leghemoglobin observed using a stereo microscope (Nikon SMZ1270). Nitrogen fixation activity of root nodules was determined using the acetylene reduction assay (67). For this, whole roots were separated from a clover plant and incubated in 5 mL glass vials containing 10% (vol/vol) acetylene in air at room temperature. After 24 h, 600 µL of the gas phase was removed from the vials and analyzed using a CarboxenTM 1010 PLOT FUSED SILICA Capillary Column [(30 m × 0.53 mm I.D. (25467)) from SUPELCO, USA] installed in a GC-2010 (Shimadzu) using the following parameters: oven 35°C (1 min) to 230°C at 24°C/min; injector temperature (SPL1): 200°C; detector temperature (TCD1): 230°C; carrier gas: helium, make-up flow: 8 mL/min. The substrate acetylene and the product ethylene were detected at a retention time of 6 and 7.5 min, respectively.

## SUPPLEMENTAL MATERIAL

Supplemental material aem.00590-24-s0001.pdf Figures S1 to S3; Tables S1 to S5.

Graphical abstract aem.00590-24-s0002.tiff Diagrammatic summary of the study.

ASM does not own the copyrights to Supplemental Material that may be linked to, or accessed through, an article. The authors have granted ASM a non-exclusive, world-wide license to publish the Supplemental Material files. Please contact the corresponding author directly for reuse.
