# Supplementary material for: Three separate pathways in Rhizobium leguminosarum maintain phosphatidylcholine biosynthesis, which is required for symbiotic nitrogen fixation with clover
Source: Appl Environ Microbiol. 2024 Aug 9;90(9):e00590-24. doi: 10.1128/aem.00590-24 (PMC11409717; doi:10.1128/aem.00590-24)
Supplement: Supplemental material — Figures S1 to S3; Tables S1 to S5. [file aem.00590-24-s0001.pdf]

Three separate pathways in *Rhizobium leguminosarum* maintain phosphatidylcholine biosynthesis, which is required for symbiotic nitrogen fixation with clover

Supplemental material

Julia Kleetz[a], Ann-Sophie Mizza[a], Irina Shevyreva[a], Leon Welter[a], Claudia Brocks[b], Anja Hemschemeier[b], Meriyem Aktas[a] and Franz Narberhaus[a, *]
[a]Microbial Biology, Faculty of Biology and Biotechnology, Ruhr University Bochum, Bochum, Germany
[b] Photobiotechnology, Faculty of Biology and Biotechnology, Ruhr University Bochum, Bochum, Germany

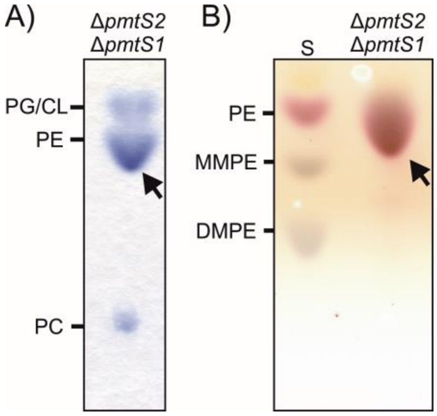

Figure S1. Lipids of *R. leguminosarum* Δ*pmtS2*Δ*pmtS1* separated by one dimensional TLC. Cells were grown in complex medium (YEM) for 3 days at 30 °C. Cultures were then harvested and lipids were extracted and separated by one dimensional TLC. (A) Phospholipids were stained with molybdenum blue reagent. (B) Free amino groups of lipids were visualized by ninhydrin staining. Arrows indicate the position of PE. S indicates the applied standard consisting of PE, MMPE and DMPE. Phosphatidylethanolamine (PE); (phosphatidylglycerol (PG); cardiolipin (CL); monomethyl-PE (MMPE); dimethyl-PE (DMPE); phosphatidylcholine (PC).

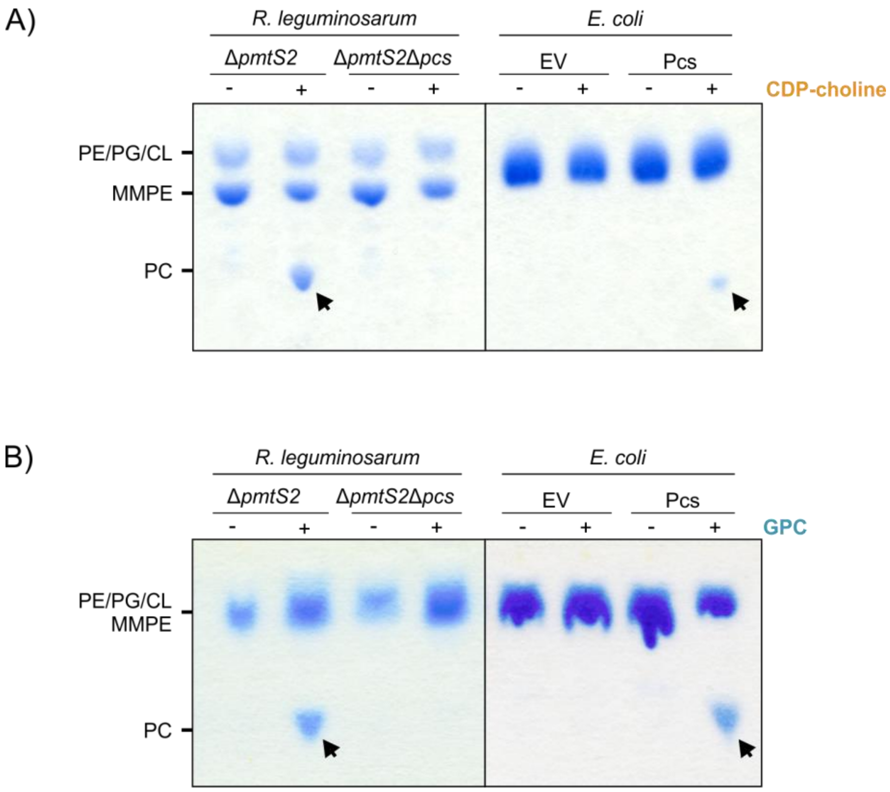

Figure S2. *R. leguminosarum* utilizes cytidine diphosphate choline (CDP-choline) and glycerophosphocholine (GPC) for PC production. The *R. leguminosarum* Δ*pmtS2* and Δ*pmtS2*Δ*pcs* mutants were cultivated in the absence (-) or presence (+) of 2.5 mM CDP-choline (A) or 1 mM GPC (B). To further elucidate the role of *R. leguminosarum* Pcs in the CDP-choline or GPC dependent PC formation, the enzyme was produced in *E. coli* BL21 (DE3). The *E. coli* BL21 (DE3) expression culture was grown in M9 minimal medium and simultaneously to gene expression induction by IPTG, CDP-choline (A) or GPC (B) was added. *E. coli* BL21 (DE3) carrying the empty vector (EV) served as negative control. Lipids were extracted, separated by TLC, and visualized using molybdenum blue reagent. The position of commercially available C18:1 phospholipids is indicated. Arrows highlight formed products.

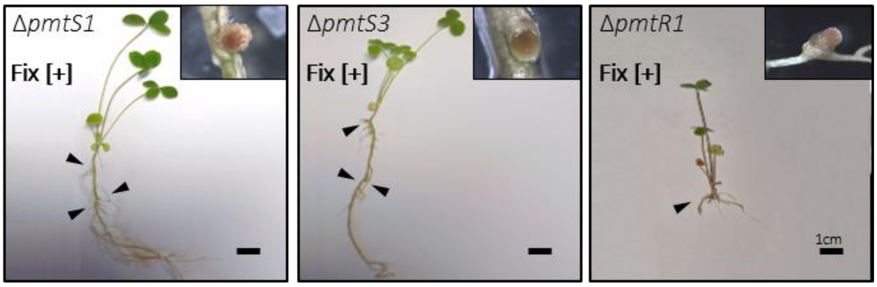

Figure S3. Representative red clover plants inoculated with *R. leguminosarum ∆pmtS1, ∆pmtS3 or ∆pmtR1* mutant strains. Clover seedlings were grown in Fahraeus agar and inoculated with *R. leguminosarum*. Plants were incubated at controlled conditions (21 °C for 16 h (day) and 17 °C for 8 h (night)) and harvested after 5 weeks. One representative plant from each condition is shown. Arrows indicate developed nodules depicted while cut open to the upper right of the respective plant. The visual appeal of the plant and the color of the cut nodules (red: with leghemoglobin, white: without) were used to assess successful or failed nitrogen fixation (Fix [+/-]).

Table S1. Strains and plasmids used in this study.

Strain or plasmid	Relevant characteristics	Source or reference
**Strain**		
Escherichia coli JM83	Cloning host	(1)
E. coli BL21 (DE3)	Host for gene expression	(2)
E. coli S17-1	Donor for biparental mating	(3)
Rhizobium leguminosarum bv. *trifolii* ATCC 14479 (also DSM 6040; 3D1K2)	Wildtype	DSMZ-German Collection of Microorganisms and Cell Cultures GmbH, Braunschweig, Germany
R. leguminosarum Δ*pmtS1*	Wildtype derivative, deletion of the *pmtS1* gene	This study
R. leguminosarum Δ*pmtS2*	Wildtype derivative, deletion of the *pmtS2* gene	This study
R. leguminosarum Δ*pmtS3*	Wildtype derivative, deletion of the *pmtS3* gene	This study
R. leguminosarum Δ*pmtR1*	Wildtype derivative, deletion of the *pmtR1* gene	This study
R. leguminosarum Δ*pcs*	Wildtype derivative, deletion of the *pcs* gene	This study
R. leguminosarum Δ*pmtS2*Δ*pmtS1*	Wildtype derivative, deletion of the *pmtS2* and *pmtS1* genes	This study
R. leguminosarum Δ*pmtS2*Δ*pmtS3*	Wildtype derivative, deletion of the *pmtS2* and *pmtS3* genes	This study
R. leguminosarum Δ*pmtS2*Δ*pcs*	Wildtype derivative, deletion of the *pmtS2* and *pcs* genes	This study
R. leguminosarum Δ*pmtS2*Δ*pcs*Δ*pmtR1*	Wildtype derivative, deletion of the *pmtS2* *pcs* and *pmtR1* genes	This study
**Plasmid**		
pET24b	Km^R, high-copy number, C-terminal 6xHis-tag expression vector	Novagen, Darmstadt, Germany
pET28b	Km^R, high-copy number, N- or C-terminal 6xHis-tag expression vector	Novagen, Darmstadt, Germany
pK19mobsacB	Km^R, mobilizable suicide vector for construction of *R. leguminosarum* mutants	(4)
pBO7067	*pmtS1* in pMA-RQ, Amp^R, GeneArt cloning vector	GeneArt, Thermo Fisher Scientific
pBO7066	*pmtS2* in pMK-RQ, Km^R, GeneArt cloning vector	GeneArt, Thermo Fisher Scientific
pBO7049	*pmtS3* in pMA-RQ, Amp^R, GeneArt cloning vector	GeneArt, Thermo Fisher Scientific
pBO7065	*pmtR1* in pMA-RQ, Amp^R, GeneArt cloning vector	GeneArt, Thermo Fisher Scientific
pBO7018	*pmtS1* from pBO7067 in pET28b, N-terminal His-tag	This study
pBO7035	*pmtS1* in pET24b, C-terminal His-tag	This study
pBO7019	*pmtS2* from pBO7066 in pET28b, N-terminal His-tag	This study
pBO7051	*pmtS3* from pBO7049 in pET28b, N-terminal His-tag	This study
pBO7034	*pmtR1* in pET24b, C-terminal His-tag	This study
pBO7017	*pmtR1* from pBO7065 in pET28b, N-terminal His-tag	This study
pBO7031	Up- and downstream region of *pmtS1* in pK19mobsacB	This study
pBO7027	Up- and downstream region of *pmtS2* in pK19mobsacB	This study
pBO7052	Up- and downstream region of *pmtS3* in pK19mobsacB	This study
pBO7050	Up- and downstream region of *pmtS3* in pMA-RQ, AmpR, GeneArt cloning vector	GeneArt, Thermo Fisher Scientific
pBO7029	Up- and downstream region of *pmtR1* in pK19mobsacB	This study
pBO7033	Up- and downstream region of *pcs* in pK19mobsacB	This study
pBO2337	pK19mobsacB derivative carrying a 3x FLAG epitope	(5)

pBO7045	pK19mobsacB including up- and downstream region of *pmtS1* with 3x FLAG epitope at 3' end	This study
pBO7040	pK19mobsacB including up- and downstream region of *pmtS2* with 3x FLAG epitope at 3' end	This study
pBO7053	pK19mobsacB including up- and downstream region of *pmtS3* with 3x FLAG epitope at 3' end	This study
pBO7038	pK19mobsacB including up- and downstream region of *pmtR1* with 3x FLAG epitope at 3' end	This study
pBO7047	pK19mobsacB including up- and downstream region of *pcs* with 3x FLAG epitope at 3' end	This study

Table S2. Oligonucleotides used in this study (Underlined sequences indicate restriction sites).

Name	Sequence (5' → 3')
**For construction of pET24b expression plasmids**	
pmtS1_fw	AAAAACATATGACTGCAAAGGCCGCATGCTCC
pmtS1_rv	AAAAAGTCGACGCCGCCGGTTTCGTT
pmtR1_fw	AAAAACATATGTCAGAGCTTCGCTACCGGGCGG
pmtR1_rv	AAAAAGTCGACGGCGGCAAGTCCGC
pcs_fw	AAAAACATATGAAGATTTTCAACTACAAGCGTG
pcs_rv	AAAAACTCGAGCTAAGCTTCGCGTCCA
**For construction of pK19mobsacB plasmids for deletion mutants**	
pmtS1-upstream_fw	AAAAAGGATCCATGGACGGGACGGATCTGT
pmtS1-upstream_rv	AAAAAGGTACCGACTATCCCACCTGTCTGAAATTC
pmtS1-downstream_fw	AAAAAGGTACCACGATGATGCCGGGCC
pmtS1-downstream_rv	AAAAAGAATTCGGCTTTCAGCGCCGGCG
pmtS2-upstream_fw	AAAAAGGATCCTGCTGATGGATATCACCGCCG
pmtS2-upstream_rv	AAAAAGGTACCTCTCCACCCATCCGCATGTTG
pmtS2-downstream_fw	AAAAAGGTACCGATGTTCGGGATCTACATCACT
pmtS2-downstream_rv	AAAAAGAATTCCGCATCATGCGTGGCGAGA
pmtR1-upstream_fw	AAAAAGGATCCCGTCTACGACAAGCAAAAGAAAACCG
pmtR1-upstream_rv	AAAAAGGTACCGCCGGCACCTCCCTAC
pmtR1-downstream_fw	AAAAAGGTACCGACCGGAAACTTTTTTCCCGT
pmtR1-downstream_rv	AAAAAGAATTCACCAGCCCGATCGCGATC
pcs-upstream_fw	AAAAATCTAGAACGCCGGCGGGGATGGA
pcs-upstream_rv	AAAAAGAGCTCATTCCCCCGACCGCCCTA
pcs-downstream_fw	AAAAAGAGCTCGATAGGCAGATGACCAAAACGC
pcs-downstream_rv	AAAAAGAATTCATTCGGCGGTCATGCCCTTCAG
**For construction of pK19mobsacB plasmids for FLAG-reporter strains**	
3xFLAG_fw	AAAAAGGATCCGACTACAAAGACCATGACGGTG
3xFLAG_rv	AAAAAGGTACCTCATTTATCGTCGTCATCTTTGTAG
pmtS1-ORF-upstream_fw	AAAAATCTAGACCCGACGGCG
pmtS1-ORF-upstream_rv	AAAAAGGATCCGCCGCCGGTT
pmtS2-ORF-upstream_fw	AAAAATCTAGACGCCATCACCAAGGCCATC
pmtS2-ORF-upstream_rv	AAAAAGGATCCGCCGCGCTTGTAGATC
pmtS3-ORF-upstream_fw	AAAAATCTAGAGCGGGATCTGACGCTGGTC
pmtS3-ORF-upstream_rv	AAAAAGGATCCTGCACGGCGCTGCT
pmtR1-ORF-upstream_fw	AAAAATCTAGAGGCGAAGCGCGAAAACCT

pmtR1-ORF-upstream_rv	AAAAAGGATCCGGCGGCAAGTCCG
pcs-ORF-upstream_fw	AAAAATCTAGACGCCGCCGGCATG
pcs-ORF-upstream_rv	AAAAAGGATCCAGCTTCGCGTCCAAGG
**For sequencing of the constructed deletion mutants**	
pmtS1-seq_fw	CGACGAAAATTCGGCGCTTCATGCCTCC
pmtS1-seq_rv	GGAGGCGCATGCGCGACCGC
pmtS2-seq_fw	CTCACCCGCCTTCATC
pmtS2-seq_rv	CGGTCGCGGGAGAT
pmtS3-seq_fw	AAGGGCTCAGCGATGCCGAC
pmtS3-seq_rv	GGCGCAAGGTTTCTGAGCGGC
pmtR1-seq_fw	GTTCGGCAGCTCGC
pmtR1-seq_rv	CGCAAAGCCCGACC
pcs-seq_fw	GAATTCGCCGGAACGGAAACGGAAGCC
pcs-seq_rv	GCCTTTGCCCACTGACCAGCGATC

Table S3. Membrane phospholipid composition of *R. leguminosarum* single deletion mutants. Relative phospholipid amounts were determined by pixel counting using the AlphaEase software. Relative amounts missing to reach 100% accounted for unidentified lipids. Quantification is based on one representative TLC for each strain and condition.

		PE [%]	PG [%]	CL [%]	MMPE [%]	DMPE [%]	PC [%]
**YEM**	**Wildtype**	4	10	11	41	10	24
	**Δ*pcs***	6	9	16	35	8	22
	**Δ*pmtS1***	11	8	13	27	7	31
	**Δ*pmtS2***	4	24	15	45	1	11
	**Δ*pmtS3***	2	12	10	42	9	25
	**Δ*pmtR1***	3	13	11	43	10	16
**YMM - cho**	**Wildtype**	4	7	10	44	9	25
	**Δ*pcs***	8	12	11	36	10	24
	**Δ*pmtS1***	13	9	11	33	8	21
	**Δ*pmtS2***	12	23	19	45	0	0
	**Δ*pmtS3***	3	9	6	44	13	26
	**Δ*pmtR1***	6	10	12	45	10	15
**YMM + cho**	**Wildtype**	3	9	11	41	8	27
	**Δ*pcs***	8	14	13	37	10	18
	**Δ*pmtS1***	17	10	8	33	6	26
	**Δ*pmtS2***	5	16	20	27	0	31
	**Δ*pmtS3***	4	12	11	44	6	23
	**Δ*pmtR1***	7	14	11	37	6	24

Table S4. Membrane phospholipid composition of *R. leguminosarum* double and triple deletion mutants. Relative phospholipid amounts were determined by pixel counting using the AlphaEase software. Relative amounts missing to reach 100% accounted for unidentified lipids. Quantification is based on one representative TLC for each strain and condition. For comparison, the Δ*pmtS2* data from Table S3 are included.

		PE [%]	PG [%]	CL [%]	MMPE [%]	DMPE [%]	PC [%]
YEM	***ΔpmtS2***	4	24	15	45	1	11
	ΔpmtS2ΔpmtS1	54	20	14	0	0	8
	ΔpmtS2ΔpmtS3	6	18	13	52	3	8
	ΔpmtS2Δpcs	4	9	17	63	2	2
	ΔpmtS2ΔpcsΔpmtR1	3	14	14	68	0	0
YMM - cho	***ΔpmtS2***	12	23	19	45	0	0
	ΔpmtS2ΔpmtS1	66	31	3	0	0	0
	ΔpmtS2ΔpmtS3	10	22	6	62	0	0
	ΔpmtS2Δpcs	15	8	13	59	0	0
	ΔpmtS2ΔpcsΔpmtR1	5	24	12	69	0	0
YMM + cho	***ΔpmtS2***	5	16	20	27	0	31
	ΔpmtS2ΔpmtS1	45	20	2	0	0	33
	ΔpmtS2ΔpmtS3	9	18	11	34	0	26
	ΔpmtS2Δpcs	12	27	3	59	0	0
	ΔpmtS2ΔpcsΔpmtR1	3	12	13	72	0	0

Table S5. Overview of *R. leguminosarum* strains with fully sequenced genomes deposited in the National Center for Biotechnology Information (NCBI) database (6).

Strain designation	biovar	Nr. of plasmids	NCBI assembly nr.	Homolog to gene from ATCC 14479				
				pmtS3	*pmtS2*	*pmtS1*	*pmtR1*	*pcs*
**ATCC 14479**	*trifolii*	4	GCA_001679785.1	+	+	+	+	+
**WSM2304**	*trifolii*	4	GCA_000021345.1	-	+	+	+	+
**CB782**	*trifolii*	3	GCA_000520875.1	-	+	+	+	+
**BIHB 1217**	*viciae*	6	GCA_002243365.1	+	+	+	+	+
**UPM791**	*viciae*	5	GCA_002948295.1	+	+	+	+	+
**23B**	*trifolii*	5	GCA_011604505.1	+	+	+	+	+
**3B**	*trifolii*	4	GCA_011604545.1	+	+	+	+	+
**4B**	*trifolii*	4	GCA_011604565.1	+	+	+	+	+
**Norway**	*viciae*	5	GCA_001679785.1	+	+	+	+	+
**TA1**	*trifolii*	4	GCA_000430465.3	+	+	+	+	+
**31B**	*trifolii*	4	GCA_011604465.1	+	+	+	+	+
**WSM1325**	*trifolii*	5	GCA_000430465.3	-	+	+	+	+
**CC275e**	*trifolii*	6	GCA_000769405.2	+	+	+	+	+
**9B**	*trifolii*	5	GCA_011604485.1	+	+	+	+	+
**3841**	*viciae*	6	GCA_000009265.1	-	+	+	+	+
**22B**	*trifolii*	5	GCA_011604525.1	-	+	+	+	+
**BIHB 1148**	*viciae*	6	GCA_002240185.1	+	+	+	+	+
**WSM1689**	*trifolii*	5	GCA_000517605.1	-	+	+	+	+
**248**	*viciae*	5	GCA_010365265.1	+	+	+	+	+
**Vaf-108**	*viciae*	8	GCA_001679785.1	+	+	+	+	+
**Vaf10**	*viciae*	7	GCA_001890425.1	+	+	+	+	+

References

1. Vieira J, Messing J. 1982. The pUC plasmids, an M13mp7-derived system for insertion mutagenesis and sequencing with synthetic universal primers. Gene 19:259–68.
2. Studier FW, Moffatt BA. 1986. Use of bacteriophage T7 RNA polymerase to direct selective high-level expression of cloned genes. J Mol Biol 189:113–30.
3. Simon R, Priefer U, Pühler A. 1983. A broad host range mobilization system for *in vivo* genetic engineering: transposon mutagenesis in Gram-negative bacteria. Nat Biotechnol 1:784–91.
4. Schäfer A, Tauch A, Jäger W, Kalinowski J, Thierbach G, Pühler A. 1994. Small mobilizable multi-purpose cloning vectors derived from the Escherichia coli plasmids pK18 and pK19: selection of defined deletions in the chromosome of Corynebacterium glutamicum. Gene 145:69–73.
5. Möller P, Overlöper A, Förstner KU, Wen T-N, Sharma CM, Lai E-M, Narberhaus F. 2014. Profound impact of Hfq on nutrient acquisition, metabolism and motility in the plant pathogen Agrobacterium tumefaciens. PLoS One 9:e110427. doi:10.1371/journal.pone.0110427
6. Database resources of the National Center for Biotechnology Information. 2018. Nucleic Acids Res 46:D8-D13. doi:10.1093/nar/gkx1095
