# Supplementary figures and images for: Three separate pathways in Rhizobium leguminosarum maintain phosphatidylcholine biosynthesis, which is required for symbiotic nitrogen fixation with clover
Source: Appl Environ Microbiol. 2024 Aug 9;90(9):e00590-24. doi: 10.1128/aem.00590-24 (PMC11409717; doi:10.1128/aem.00590-24)

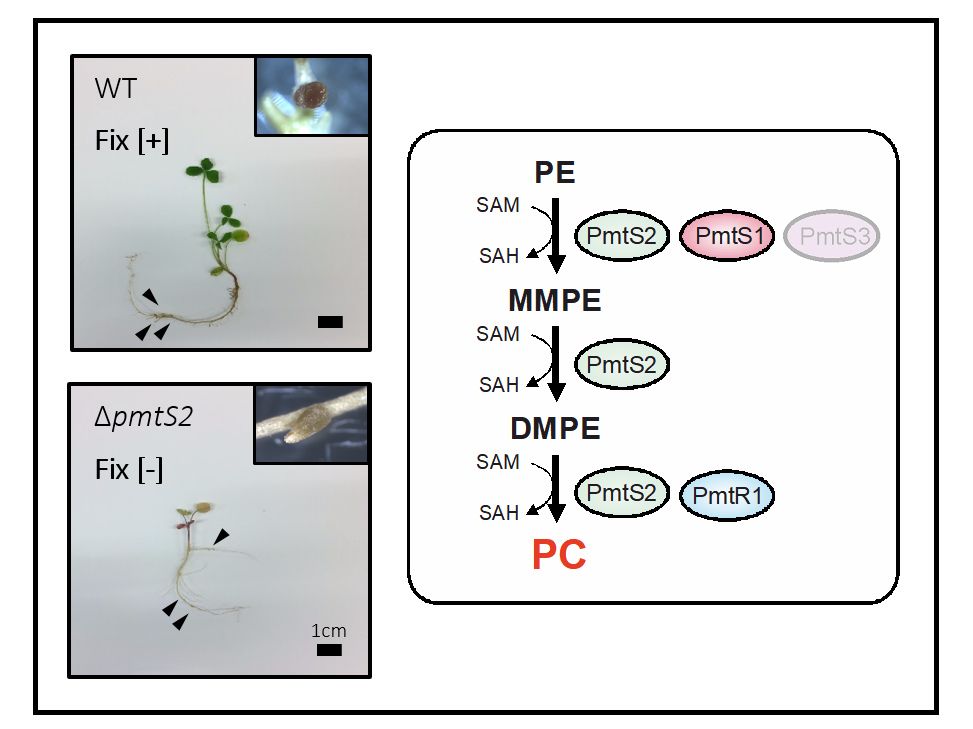

Supplement: Graphical abstract — Diagrammatic summary of the study. [file aem.00590-24-s0002.tiff]
